# Targeting the IL-17C-M2 macrophage axis ameliorates fibrosis in endometriosis through MAPK/ERK signaling

**DOI:** 10.1186/s12967-026-08769-9

**Published:** 2026-08-05

**Authors:** Jingling Li, Bo Yao, Liuying Chu, Gendie E. Lash, Qinsheng Lu, Minghua Wang, Hong Zhou, Xuefeng Ma, Ping Li

**Affiliations:** 1 Department of Pathology, Jinan University School of Medicine, Guangzhou, 510632 China; 2 Department of Pathology, Jinan University First Affiliated Hospital, Guangzhou, 510632 China; 3 Institute of Reproductive Health and Perinatology, Guangzhou Women and Children’s Medical Center, Guangzhou Medical University, Guangzhou, 510632 China; 4 Department of Pathology, Longgang District People’s Hospital, The Second Affiliated Hospital of The Chinese University of Hong Kong, Shenzhen, 518172 China; 5 Department of Obstetrics and Gynecology, Jinan University First Affiliated Hospital, Guangzhou, 510632 China; 6 Department of Obstetrics and Gynecology, Fifth Affiliated Hospital of Jinan University (Shenhe People’s Hospital), Heyuan, 517000 China

**Keywords:** Endometriosis, Macrophage, Endometrial stromal cell, Fibrosis, IL-17 C, IL-17RE

## Abstract

**Background:**

Endometriosis is characterized by inflammation and fibrosis, in which cytokines and cytokine-macrophage interactions serve as critical mediators. However, the specific mechanisms that initiate the fibrotic cascade in endometriosis remain poorly defined.

**Methods:**

Spatial transcriptomics (ST) and cellular interaction analyses were performed on human specimens of endometriotic lesions and normal endometrium. An endometriosis mouse model was used to evaluate the effect of IL-17 C neutralization with the MOR106 antibody on ectopic lesion growth and fibrosis. In vitro experiments were conducted to explore the role of IL-17 C in macrophage polarization and extracellular matrix (ECM) production by endometrial stromal cells (ESCs).

**Results:**

IL-17RE expression was significantly upregulated in endometriotic tissues compared with normal endometrium and correlated with endometriosis fibrosis. IL-17 C levels were markedly higher in patient-derived tissues and peritoneal fluid. In a mouse model of endometriosis, neutralization of IL-17 C with the MOR106 antibody inhibited ectopic lesion growth and alleviated fibrosis in both eutopic and ectopic endometrium. Importantly, MOR106 selectively reduced CD206 + M2 macrophage infiltration in lesions without significantly altering the M1 macrophage population, suggesting that IL-17 C primarily drives M2-like macrophage accumulation in vivo. In vitro, IL-17 C promoted macrophage polarization toward a pro-fibrotic M2-like phenotype, and these IL-17 C-induced M2 macrophages enhanced ECM production by ESCs via activation of the MAPK/ERK pathway.

**Conclusions:**

Targeting the IL-17 C/IL-17RE axis provides a promising novel therapeutic strategy for mitigating fibrosis in endometriosis.

**Supplementary Information:**

The online version contains supplementary material available at 10.1186/s12967-026-08769-9.

## Background

Endometriosis is the most prevalent benign gynecologic disease that affects approximately 5–15% of reproductive-aged women globally [1, 2]. It manifests as pelvic pain, dysmenorrhea, dyspareunia, subfertility, and an increased cancer risk [3]. Asian women exhibit the highest global prevalence [4], with 40 million cases in China, and its socioeconomic burden is substantial [5]. Current therapeutic strategies, primarily hormonal suppression and invasive surgery, suffer from > 50% adverse event rates and high recurrence, largely attributed to unresolved fibrosis and dysregulated inflammation [1, 6].

The presence of endometrial stroma and glands in ectopic sites is currently the main criterion of histopathologic diagnosis of endometriosis. However, fibrosis is a pathological hallmark across all endometriosis subtypes [4]. In 40% of ovarian endometriomas, the inner surface of the cyst is covered only by fibrotic tissue [7]. In peritoneal endometriosis, pelvic adhesions are typically fibromuscular tissue without any endometrial components [3]. Indeed, fibrosis is progressive over time and may be an end stage of endometriosis. Emerging evidence implicates dysregulated cytokine networks in driving both fibrosis and disease progression [8, 9]. The interleukin-17 (IL-17) family, from IL-17 A to IL-17 F, plays a critical role in pathogenesis of various inflammatory diseases, with both pro-fibrotic effects on fibroblasts and potential anti-fibrotic effects that limit ECM degradation [10]. IL-17 A is classically produced by immune cells, and has been studied mainly as an immune-derived pro-inflammatory cytokine in endometriosis [11]. In contrast, IL-17 C is mainly produced by epithelial and stromal cells, suggesting a lesion-localized mechanism distinct from the classical immune-cell-derived IL-17 A pathway [12]. Specifically, IL-17 C signaling via the unique receptor complex IL-17RE/IL-17RA is induced early in the pathogenesis and promotes cell survival in tumors [13–15]. In addition, IL-17 A and IL-17 C levels in peritoneal fluid correlate with the severity of endometriosis [13, 16], while elevated IL-17RE in peritoneal fluid strongly associates with advanced-stage endometriosis, particularly in endometriomas [13]. Although the IL-17 C inhibitor MOR106 shows promise in Phase Ⅰ/Ⅱ trials for inflammatory fibrotic diseases [17], its role in endometriosis-related fibrosis remains uncharacterized.

Endometrial-resident macrophages comprise approximately 15–20% of leukocytes [18], with profound infiltration and an increased M2:M1 polarization ratio being defining features of endometriotic lesions [19], which correlates with fibrosis severity in preclinical models [20–23]. Indeed, these macrophages exhibit a ‘pro-endometriosis’ phenotype and are frequently found in the peritoneal lining [21, 24]. Endometriosis-associated fibrosis, characterized by excess deposition of extracellular matrix (ECM) components, is orchestrated through dysregulated cytokine networks and aberrant macrophage polarization. Myofibroblasts serve as pivotal effector cells in this process, driving pathological ECM overproduction [25]. Evidence indicates that endometriosis progression hinges on cytokine-macrophage crosstalk, which subsequently activates myofibroblasts and disrupts ECM homeostasis [26].

Recent advances in single-cell RNA sequencing and spatial transcriptomics (ST) have substantially improved the ability to identify therapeutic biomarkers by resolving cellular heterogeneity and preserving tissue spatial organization [27, 28]. In the current study, ST analysis in endometriosis demonstrated colocalized populations of lesion-resident M2-like macrophages and fibrotic niches, identifying the IL-17 C/IL-17RE as mediators of macrophage-fibroblast interactions. Additionally, the effects of IL-17 C neutralization were investigated in vivo endometriosis models, in vitro M2-polarized macrophage (M2-THP1 cells), and primary endometrial stromal cells (ESCs) co-cultures. These studies aimed to dissect the cellular and molecular mechanisms contributing to the development of fibrosis in endometriosis and define the critical factors for macrophage-fibroblast crosstalk, potentially enabling dual cell-specific therapeutic targeting.

## Methods

### Patients and tissue sample collection

For the ST analysis, normal endometrium and endometrioma tissues were collected from patients who underwent surgical resection. Each sample was split into 3 parts for performing frozen sections for histological diagnosis, formalin fixed and paraffin-embedded sections for immunohistochemistry analysis, and stored at − 80 °C for other analyses. The clinical characteristics of enrolled subjects are summarized in Supplementary Table S1. For validation experiments, patients with endometriosis (*n* = 28) were recruited, and peritoneal fluid and tissue samples were collected. Normal endometrium and peritoneal fluid samples (*n* = 25) were obtained from age-matched women undergoing interval tubectomy. The clinical characteristics of enrolled subjects are summarized in Supplementary Table S2. Premenopausal women who underwent laparoscopic surgery were enrolled in this study. The exclusion criteria were irregular menstrual cycle, hormone use within 3 months prior to surgery, pregnancy, malignancy, autoimmune or inflammatory diseases, or other gynecological disorders that could affect endometrial status. All endometriosis cases and control endometrial samples were confirmed by laparoscopic surgery and postoperative histological examination.

### Spatial transcriptomics sequencing and cellular interaction analyses

Human biopsy tissues less than 1 cm × 1 cm in size were snap-frozen, embedded in Optimal Cutting Temperature (O.C.T.) compound, and stored at − 80 °C. Total RNA was extracted from these frozen embedded tissues using standard methods to ensure high integrity. Resulting tissue blocks were cryosectioned at a thickness of 10 μm and processed for STseq (10x Genomics Visium). For quality control, genes detected in fewer than 0.1% of all cells were excluded. Low-quality cells were removed if they had fewer than 200 detected features or a mitochondrial gene fraction greater than 10%. Potential doublets were identified using DoubletFinder and excluded before downstream analysis. Raw unique molecular identifier (UMI) counts were normalized with SCTransform, and the top 3,000 variable features were selected for subsequent analyses. After quality control filtering, the final Seurat objects contained a total of 2648–3848 spots per tissue, and each spot contained approximately 50-153761 unique molecular identifiers (UMIs). Specifically, normal tissue contained 2648 spots and 9810 genes, endometrioma tissue contained 3848 spots and 7690 genes. These sample-level dataset statistics are also summarized in Supplementary Figure S1.

Potential batch effects among samples were corrected using Harmony integration implemented in the Seurat package (version 5.2.0) [29]. Subsequent analyses included dimensionality reduction via Uniform Manifold Approximation and Projection (UMAP) and cell clustering using Seurat’s FindClusters function with a resolution parameter of 0.8. Cell clusters were preliminarily annotated based on the expression patterns of highly variable genes and known marker genes. Cell type annotation was performed using the following marker genes: endometrial gland: EPCAM, CDH1; endometrial luminal epithelium: EPCAM, K18, ESR1; fibrotic stroma: ACTA2, FN1, COL1A1; Immune cell: CD79A, CSF3R, IL2RB, GZMM; endometrial stroma: COL6A1, VIM; ovarian stroma: SVIL, PRELP; blood vessel: MCAM, CLDN5; blood Area: PLAUR, TXLNA; CD206 + macrophage: CD68, CD14, CD163, MRC1; CD86 + macrophage: CD68, CD14, CD163, CD86; fibrotic stroma-1: VIM, COL6A1; fibrotic stroma-2: ACTA2, COL6A1; T-B cell: CD79B, MZB1, CD79A, CD3E, CD3D, TRAC; smooth muscle cell: RGS5, MCAM, PDGFRB; endothelial cell: PECAM1, CD34, ESAM, CLDN5. These annotations were subsequently reviewed and refined by a pathologist to confirm their correspondence with the histological regions observed on the tissue sections. Kyoto Encyclopedia of Genes and Genomes (KEGG) enrichment analysis was performed using the clusterProfiler package (version 4.16.4). Gene Set Variation Analysis (GSVA) for Gene Ontology (GO) terms was implemented via the gsva package (version 2.2.0) [30].

### Trajectory analysis

Trajectory analysis was performed using Monocle 2 (version 2.32.0). Cells of interest were extracted from the Seurat object and converted into a CellDataSet object using the raw count matrix. Size factors and gene dispersions were estimated using the estimateSizeFactors and estimateDispersions functions, respectively. Genes expressed at a minimum expression level of 0.1 were retained for downstream analysis. Dimensionality reduction and trajectory construction were performed using the DDRTree algorithm implemented in the reduceDimension function [31].

### Animal experiments

Eight-week-old female C57BL/6J mice were obtained from Beijing Vital River Laboratory Animal Technology Co., Ltd (China). All mice were housed in a controlled environment at 22 ± 3 °C and 35 ± 5% humidity, under a 12 h/12 h light/dark cycle. Vaginal smears were performed daily to monitor the estrous stage. Only mice exhibiting regular estrous cycles were selected for the endometriosis modeling procedure. To establish the endometriosis mouse model, donor mice (*n* = 10) received an intramuscular injection of 17β-estradiol-3-benzoate (100 µg/mouse; Cat# E8515, Sigma-Aldrich). One week after injection, donor mice were sacrificed. The uteri were excised, and the two uterine horns were isolated. The myometrium was then scraped off to isolate the endometrium. The endometrial tissue was rinsed twice with PBS and minced into fragments measuring 3–5 mm³. Approximately equal-sized endometrial fragments were then transplanted intraperitoneally into recipient mice. Endometrial tissue from one donor mouse was transplanted to two recipient mice. Subsequent to transplantation, recipient mice received subcutaneous injections of 17β-estradiol-3-benzoate (100 µg/mouse) every three days. Two weeks post-transplantation, establishment of endometriotic lesions was confirmed. Mice bearing confirmed lesions were then randomly assigned to different treatment groups using a random number table (*n* = 10 per group). Because of the nature of the intervention, blinding was not feasible during treatment administration; however, lesion assessment and data analysis were performed by investigators blinded to group allocation. MOR106 (BR2010231, Shanghai BioLeaper Biotechnology Co., Ltd., China) was administered intraperitoneally at a dose of 10 mg/kg, twice weekly for one week. Upon sacrifice, ectopic lesions were excised, weighed, and fixed and embedded in paraffin for subsequent histological analyses.

### Enzyme-linked immunosorbent assay (ELISA)

Peritoneal fluid or cyst fluid samples were collected from human patients during surgical procedures. For mouse experiments, peritoneal lavage fluid was obtained by intraperitoneal injection of 1 mL PBS. Prior to cytokine measurements, the murine peritoneal lavage fluid was centrifuged at 1,500 × g for 5 min. The resulting supernatant was collected, and a 500 µL aliquot was used for the analysis. Concentrations of IL-17 C in the human and murine samples were measured using commercial ELISA kits: human IL-17 C (BS-E3942H2) and mouse IL-17 C (BS-E10643M2; both from Jiangsu Boshen Biotechnology Co., Ltd., Jsbossen, China). Additionally, inflammatory factors tumor necrosis factor-alpha (TNF-α), interleukin-6 (IL-6), and interleukin-1β (IL-1β) (#JL10484; #JL20268; #JL18442 from JONLBIO Co., Ltd., Shanghai, China) were assessed in the murine peritoneal fluid and serum samples. All assays were performed strictly in accordance with the manufacturer’s instructions. Both intra-assay and inter-assay coefficients of variation were documented to be less than 5%. Absorbance was measured at 450 nm using a microplate reader (TZCAN-SAFIRZ-Z, TZCAN, Austria).

### Histology assessment

Tissue samples were fixed, routinely embedded in paraffin, and sectioned (5 μm thick) and stained with hematoxylin and eosin (H&E) for routine histological evaluation. Fibrosis was specifically assessed using Masson’s trichrome (Masson staining) and Sirius Red. Immunohistochemistry (IHC) and immunofluorescence (IF) staining were performed according to established protocols described previously [25]. Stained sections were examined and photographed using an epifluorescence microscope (Olympus IX51, Leica DM 4000B).

Quantitative analysis was performed using ImageJ software (version 1.54j). For Masson’s trichrome and Sirius Red staining, the extent of fibrosis was quantified by measuring the positively stained areas. For IHC staining, the staining intensity of specific markers was quantified using ImageJ. For each tissue sample, values from selected sections were averaged to obtain one representative value per tissue. A systematic random sampling strategy was used for section selection, with every fifth and sixth sequential histological section from each tissue block selected for comparative analysis and evaluation (*n* = 5 tissues per group).

### Isolation and culture of primary human endometrial stromal cells (ESCs)

Primary human endometrial stromal cells (ESCs) were isolated and prepared as described previously [32]. Briefly, endometrial tissue biopsies were obtained during the proliferative stage of the menstrual cycle from healthy women (*n* = 6, aged 24–40 years). Tissue samples were immediately rinsed in sterile PBS and minced into approximately 1–2 mm³ fragments using sterile surgical scalpels. Minced tissue fragments were subjected to enzymatic digestion at room temperature with constant agitation for 30 min. The digestion solution consisted of Collagenase I (1 mg/mL, Sigma-Aldrich, St. Louis, MO, USA) and DNase I (1 mg/mL, Gibco) in PBS. The digested tissue suspension was sequentially filtered through a sterile 150 μm nylon mesh cell strainer to remove undigested debris. The filtrate containing smaller cell clusters and single cells was then passed through a 40 μm sterile cell strainer. This filtration step retains epithelial cell clumps while allowing the smaller, individual stromal cells to pass through. The cells were centrifuged at 200 g × 5 min. The supernatant was discarded and the cells were resuspended and cultured in DMEM/F12 complete medium. Cellular phenotype was also confirmed by immunocytochemistry staining with anti-vimentin antibodies (Cell Signaling Technology, USA). The purity of isolated stromal cells was more than 95% (Supplementary Figure S2).

### Cell culture and treatment

The THP-1 cells were obtained from ProCell Corporation (Wuhan, China) and cultured in RPMI 1640 medium and DMEM/F12 (Gibco, Massachusetts, USA) (5% CO_2_, 20% O_2_, 37 °C). Moreover, the effects of MOR106 (BR2010231, Shanghai Bioleaper Biotechnology Co. Ltd., China) were evaluated in cells. M2 macrophages can be induced by phorbol-12-myristate-13-acetate (PMA) and IL-4 in vitro. The cells were treated with 100 ng/ml PMA (Sigma, 16561, USA) for 24 h, then with 30 ng/ml IL-4 (PeproTech, 200-04, USA) for an additional 48 h to generate M2-like macrophages.

For coculture, 0.4 μm transwell inserts (Corning, NY, USA) were used. A total of 5 × 10^4^ THP-1 cells/ml were seeded into the upper chambers of a 6-well plate, and 2 × 10^4^ ESCs/ml were seeded into the lower chambers of a 6-well plate, the ERK inhibitor U0126 (S1901, Beyotime, Shanghai, China) and p38MAPK inhibitor SB203580 (S1076, Selleck, USA) were evaluated in cells. All in vitro cell experiments were performed using three independent biological replicates unless otherwise stated, and each experimental condition was assessed in technical triplicate.

### qRT-PCR and western blot analysis

qRT-PCR and Western blot were performed as previously described [33]. The specific primers and antibodies are presented in Supplementary Tables S4 and S5. The expression of genes was normalized to GAPDH. For qRT-PCR analysis, each biological replicate was measured in technical triplicate. Western blot experiments were independently repeated three times, and representative blots from three independent experiments were shown.

### Statistics

All analyses were performed using GraphPad Prism 9.0 Software (GraphPad Software, San Diego, CA). Continuous data are expressed as mean ± standard deviation (SD) for normally distributed data or as the median with interquartile range (IQR) for non-normally distributed data. Categorical variables are presented as number. IHC intensity data are shown as mean intensity per area in arbitrary units. A two-sided *P* < 0.05 was considered statistically significant.

Normality of data distribution for continuous variables was assessed using the Kolmogorov-Smirnov test. For normally distributed data with equal variance, differences between two independent groups were analyzed using the unpaired Student’s *t*-test. For non-normally distributed data, the Mann-Whitney U test was used. Differences among three independent groups were analyzed using one-way analysis of variance (ANOVA) for normally distributed data, or Kruskal-Wallis test for non-normally distributed data, followed by appropriate post-hoc tests when significant differences were found. Associations between clinicopathologic categorical variables and IL-17 level were evaluated using the Chi-square χ² test, as appropriate.

## Results

### Immune infiltration and fibrosis in endometriotic lesions

**Fig. 1:**
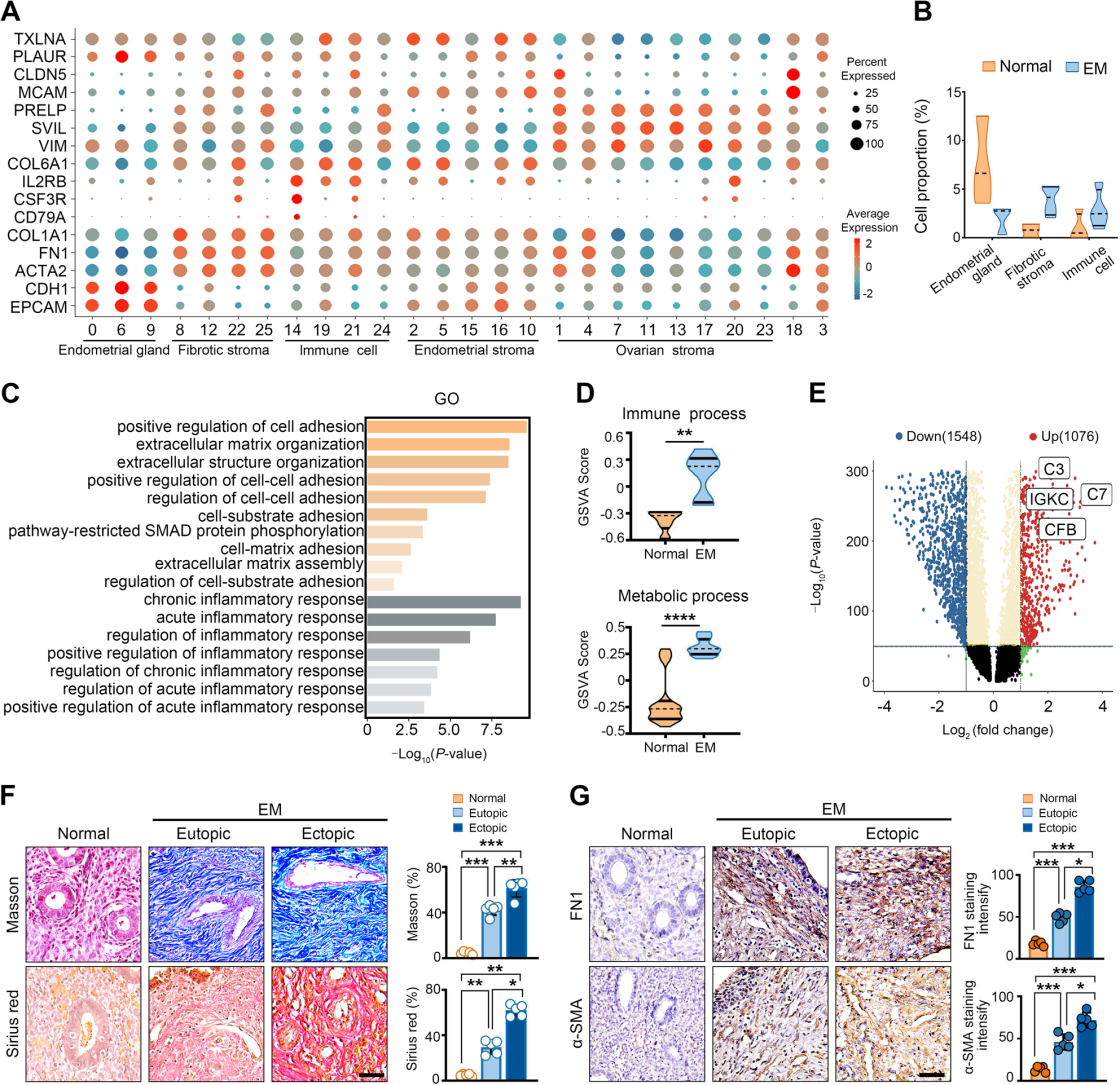
Pathological remodeling in endometriotic lesions characterized by immune dysregulation and fibrosis. (**A**) Dot plot visualization validating the identity of spatially-resolved endometrial gland, fibrotic stroma, and immune cells based on lineage-specific marker gene expression in EM lesions and normal endometrium. (**B**) Quantitative comparison of cell type frequencies and assessment of cellular spatial abundance. (**C**) Functional enrichment for ST data in EM lesions vs. control. (**D**) GSVA score. (**E**) Volcano plot of upregulation of immune-related genes in EM lesions. (**F**) Fibrosis assessment by Masson’s trichrome (blue) and Sirius Red (red) staining. (**G**) Immunohistochemical staining of ECM-related proteins (FN1 and α-SMA). Scale bars: 50 μm. *P < 0.05, **P < 0.01, ***P < 0.001 and ****P < 0.0001 for the indicated comparisons. EM, endometriosis

To delineate the cellular composition and transcriptional landscape of endometriotic lesions (EM), we performed an in-depth analysis of previously published spatial transcriptomics (ST) data. Initial histological characterization using established markers (E-cadherin staining for epithelial cells and α-SMA for myofibroblasts) confirmed distinct tissue compartments within the lesions (Supplementary Figure S3). Based on this spatial annotation, we successfully isolated transcriptomic profiles corresponding to epithelial cells, fibroblasts, and macrophages from the ST data. Dot plot analysis of established lineage-specific marker genes confirmed the identity of these isolated cellular compartments (Fig. 1A). Quantitative assessment of cell type frequencies within EM lesions revealed significant alterations compared to control endometrium. EM lesions exhibited a decreased proportion of epithelial cells, alongside an increase in fibroblasts. Furthermore, the spatial organization and/or abundance of immune cells appeared to be increased in EM lesions (Fig. 1B). Global gene expression patterns differed substantially between EM lesions and control endometrium (Pearson correlation coefficient = 0.405, *P* < 2 × 10^− 16^; Supplementary Figure S4). Gene Ontology (GO) terms showed significant enrichment in biological processes of ECM organization, cellular adhesion, and inflammatory response (Fig. 1C). Consistent with this, Gene Set Variation Analysis (GSVA) scores indicated elevated immune responses and dysregulated metabolic processes in EM compared to control tissue (Fig. 1D). Importantly, transcript levels of immune-related genes were markedly elevated in EM lesions, with genes such as *C3*, *C7*, *IGKC and CFB* displaying increased expression (Fig. 1E). Complementing the GO enrichment findings pointing to ECM dysregulation, Masson’s trichrome and Sirius Red staining demonstrated significantly increased fibrotic areas and collagen deposition in ectopic lesions compared to both eutopic and control endometrium (Fig. 1F). This fibrotic phenotype was supported by immunohistochemistry, which showed significantly upregulated protein expression of key ECM components in EM lesions, including fibronectin 1 (FN1) and α-SMA (Fig. 1G). While recent studies suggest that the endometriotic stroma is minimally altered compared to eutopic endometrium [22, 34], our findings demonstrate that ectopic endometriotic stroma undergoes substantial pathological remodeling, encompassing both cellular and ECM dysregulation.

### Spatial co-localization of M2-like macrophages and fibroblasts, linked to IL-17 C signaling and fibrosis

**Fig. 2:**
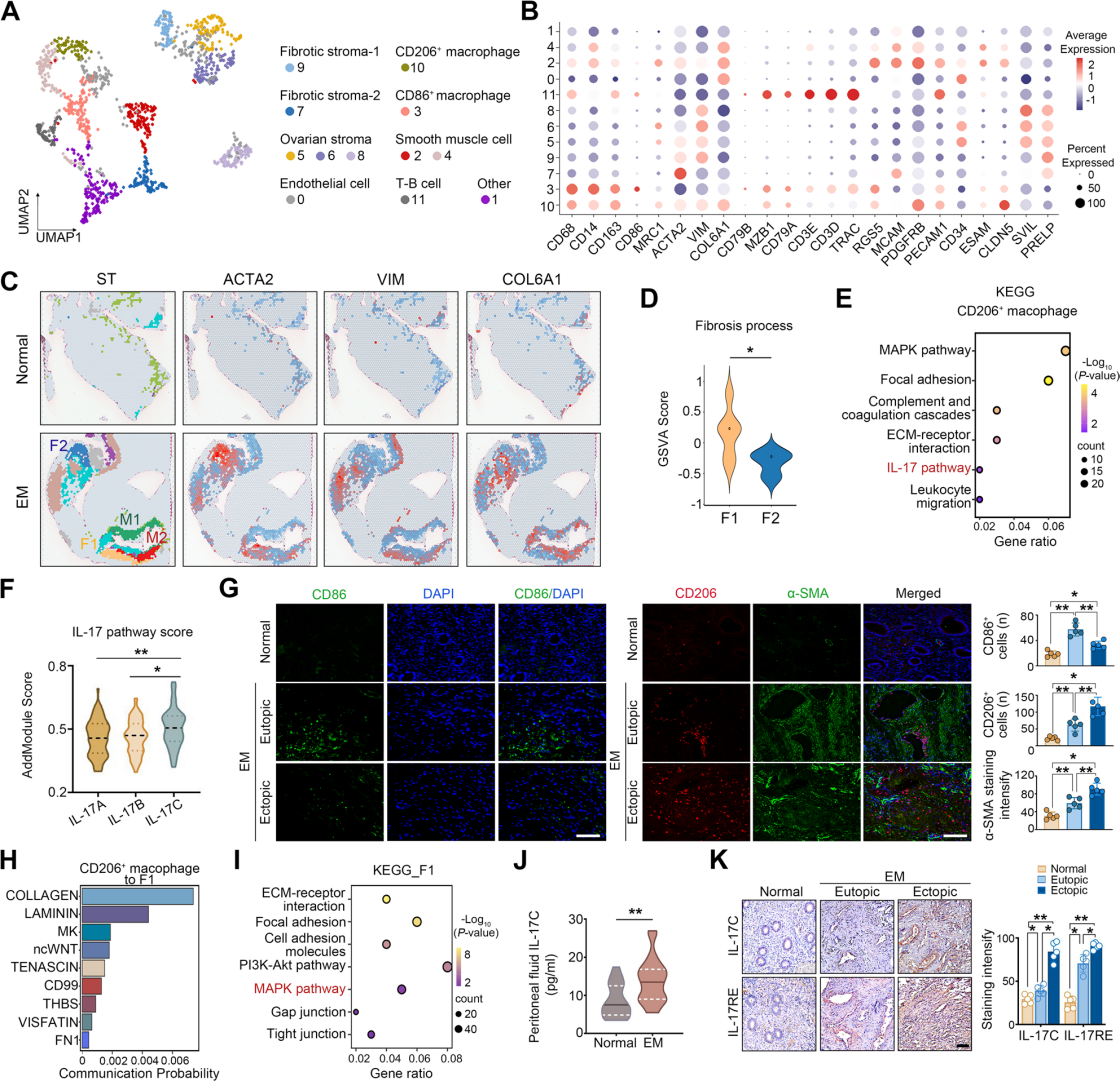
Spatial characterization reveals M2-like macrophage–fibroblast co-localization and IL-17 C-driven fibrosis in endometriosis. (**A**) UMAP of ST data showing major stromal cell types identified in normal endometrium and endometriosis lesions. (**B**) Dot plot validating stroma clusters of the two tissues ST data. (**C**) Overlay of M2-like macrophage Cluster 10 and fibroblasts F1 locations. (**D**) GSVA score of fibrosis process. (**E**) KEGG pathway enrichment analysis in macrophage cluster 10. (**F**) AddModule score. (**G**) Immunofluorescence staining for CD86, CD206 and α-SMA. (**H**) Predicted cell-cell interaction networks identifying enriched signaling pathway between co-localized M2-like macrophages and fibroblasts F1. (**I**) KEGG pathway enrichment analysis in F1 fibroblasts. (**J**) ELISA of IL-17 C concentrations in the peritoneal fluid. (**K**) Immunohistochemical analysis of IL-17 C and IL-17RE.Scale bars: 50 μm. *P < 0.05 and **P < 0.01 for the indicated comparisons. ST, Spatial transcriptomic

To investigate stroma alterations in endometriosis, we analyzed ST data and classified multiple stromal cell type populations, including fibroblast, macrophage, smooth muscle cell (SMC), endothelial cell (EC), T cell and B cell (Fig. 2A). Clusters 3 and 10 showed high expression of macrophage markers (*CD68*, *CD14*, *CD163*, *CD86* and *MRC1*), indicating macrophage-enriched endometrial stroma (Fig. 2B). These macrophages were predominantly located in endometriotic lesions, with scattered distribution in normal tissues (Fig. 2C). Notably, M2-like macrophages (CD206^+^) were spatially colocalized with a fibroblast subcluster (Fibrotic stroma 1, F1) highly enriched in ectopic lesions (Fig. 2B and C). A high density of α-SMA, a hallmark of myofibroblast phenotype [35], was detected in macrophage (CD206^+^) regions, supporting a role for macrophage-fibroblast crosstalk in fibrotic activation. GSVA analysis confirmed a significantly greater fibrosis score in fibroblasts (F1) than fibrotic stroma 2 (F2), while fibroblasts F1 were more proximal to macrophages compared with those in F2 (Fig. 2D), suggesting a microenvironment favoring fibroblast recruitment, phenotypic transition, and localized fibrosis. KEGG enrichment analysis of Cluster 10 (CD206^+^ macrophage) revealed strong activation of the IL-17 signaling pathway, with the IL-17 C pathway specifically upregulated in macrophages within endometriotic lesions (Fig. 2E and F).

In addition, ST analyses revealed that IL-17RE was enriched in macrophage-associated regions and localized within a distinct inflammatory microenvironment. To further characterize the dynamic state of these cells, pseudotime trajectory analysis was performed on macrophage populations. The results showed that IL-17RE expression gradually increased along the inferred trajectory, indicating that IL-17 C/IL-17RE signaling may participate in a progressive macrophage activation program (Supplementary Figure S5).

To assess the generalizability of our results, we performed an external validation using the GSE179640 single-cell dataset. Consistent with our initial findings, the validation analysis showed an expansion of macrophage and fibroblast populations and a reduction in the epithelial fraction in ovarian EM lesions relative to controls (Supplementary Figure S6A–C). KEGG enrichment analysis further highlighted the activation of the IL-17 signaling pathway, specifically through the upregulation of IL-17RE (Supplementary Figure S6D and E). Collectively, these data demonstrate the high reproducibility and reliability of our discovery cohort results.

Double immunofluorescence staining showed altered macrophage polarization: in both eutopic and ectopic endometrium, both CD86 and CD206 expression were increased (Fig. 2G). In ectopic lesions, both CD206 and α-SMA expression were elevated, and enrichment of CD206⁺ macrophages was observed in fibroblast-rich areas, suggesting either α-SMA acquisition by macrophages or fibroblast activation contributing to fibrosis [35].

To delineate communication between macrophages and fibroblasts, we analyzed intercellular signaling within lesions. Fibroblast clusters F1 expressed ECM molecules such as *COLLAGEN*, *THBS*, *LAMININ*, *FN1* (Fig. 2H), consistent with their roles in fibrosis [25, 36]. KEGG enrichment analysis of Fibroblast clusters F1 revealed enrichment of MAPK/ERK signaling, highlighting their relevance in fibrogenic signaling (Fig. 2I and Supplementary Figure S7).

Consistent with these findings, ST revealed minimal IL-17 C expression in normal endometrium, but markedly increased stromal IL-17 C and IL-17RE in endometriosis (Supplementary Figure S8). In line with the critical role of peritoneal fluid in disease pathology [16, 37], ELISA showed that IL-17 C levels were significantly elevated in peritoneal fluid from patients with endometriosis (*n* = 22) compared to controls (*n* = 20) (*P* = 0.01) (Fig. 2J). Immunohistochemistry confirmed the absence of IL-17 C/IL-17RE in normal endometrial stroma, but significant upregulation in ectopic lesions compared to both normal and eutopic endometrium (*n* = 5 per group) (Fig. 2K).

### Neutralization of IL-17 C attenuates fibrosis and inhibits the MAPK/ERK pathway in the ectopic endometrium of mice

**Fig. 3:**
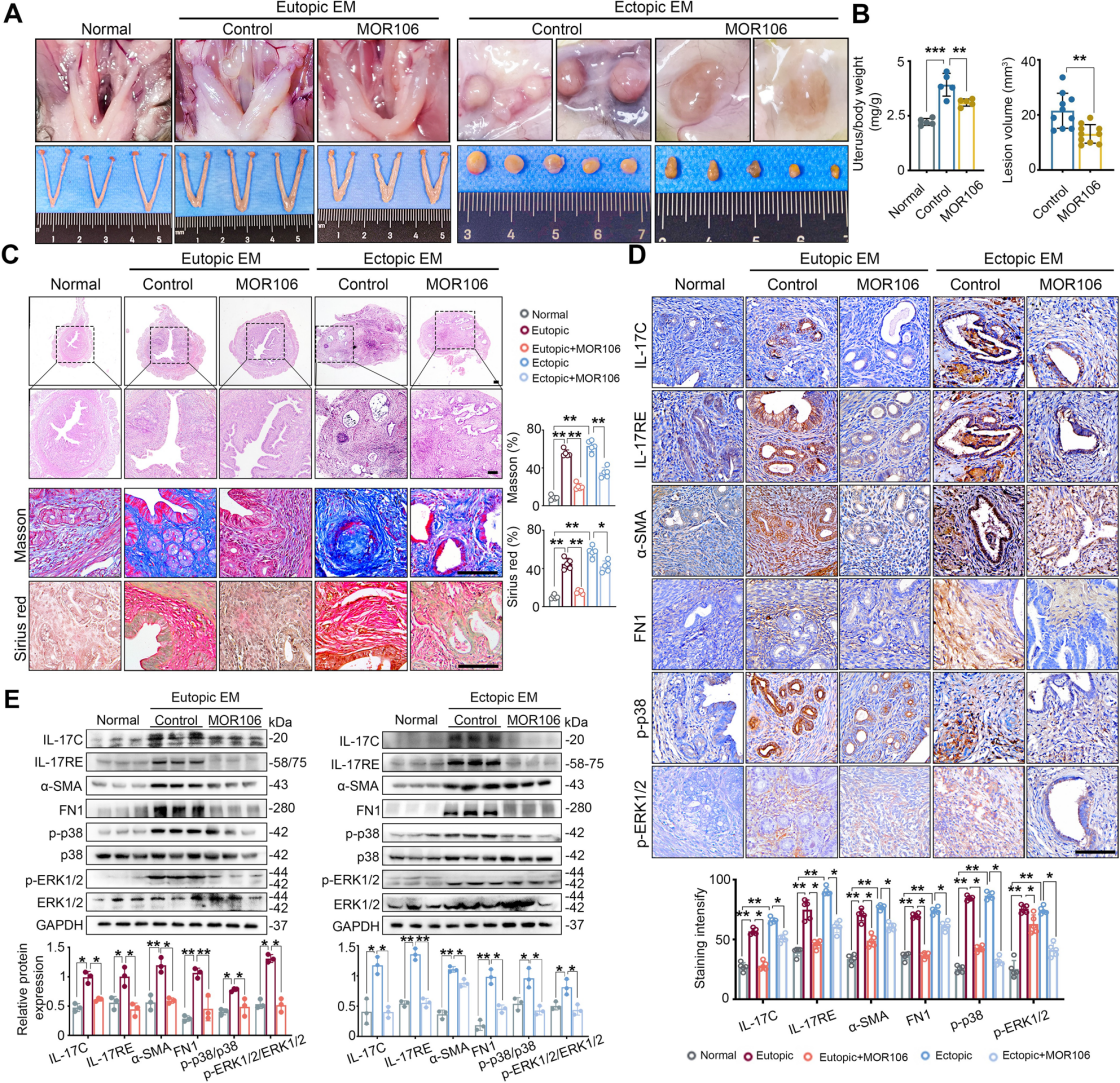
MOR106 reduces lesion burden and fibrosis in a mouse model of endometriosis. (**A**) Representative macroscopic observation. (**B**) Quantification of ratio of uterus-to-body weight, and endometriotic lesions of mice. Data represent the mean ± SD (*n* = 5). (**C**) The H&E staining and Masson’s trichrome and Sirius Red staining. (**D** and **E**) Immunostaining and western blot of IL-17 C, IL-17RE, ECM-related and MAPK/ERK pathway key proteins. Scale bars: 100 μm. *P < 0.05, **P < 0.01 and ***P < 0.001 for the indicated comparisons. EM, endometriosis

To investigate the role of IL-17 C in vivo, we utilized an established mouse model of endometriosis. Specifically, endometrial fragments were transplanted into the peritoneum of female C57BL/6J mice and allowed to develop for two weeks. These endometriosis-induced mice were then treated with either the MOR106 antibody at 10 mg/kg, which neutralizes IL-17 C and blocks the IL-17 C/IL-17RE interaction in both humans and mice [38], or an isotype IgG control antibody (Supplementary Figure S9). No significant differences in body weight were observed between the treatment groups throughout the experimental period (Supplementary Figure S10). Peritoneal implantation of endometrial fragments induced marked changes in uterine morphology in the EM group. Compared to controls, uteri in the EM group exhibited swelling and thickened walls (Fig. 3A), indicative of inflammation or irritation. Quantification revealed a significant increase in the uterus to body weight ratio in the EM group relative to controls (Fig. 3B). MOR106 administration significantly reduced uterus to body weight ratio and the size of endometriotic lesions in the peritoneal cavity (Fig. 3B). H&E staining showed treatment with MOR106 ameliorated these EM-induced morphological alterations in endometrial glands. Masson trichrome and Sirius Red staining demonstrated that MOR106 treatment significantly reduced fibrosis within both the eutopic and ectopic endometrial tissues (Fig. 3C).

To elucidate the underlying mechanism, we analyzed key molecular markers. Immunohistochemistry and western blot analyses revealed that MOR106 decreased the expression of IL-17 C and IL-17RE, along with ECM-related proteins (FN1 and α-SMA). MOR106 treatment also suppressed the activation of the MAPK/ERK pathway, as evidenced by reduced phosphorylation of p38MAPK and ERK1/2 in both eutopic and ectopic endometrial tissue (Fig. 3D and E). These findings demonstrate that neutralization of IL-17 C using MOR106 ameliorates key pathological features of EM, including uterine inflammation, lesion development, and fibrosis. Our results further indicate that the MAPK/ERK pathway, previously implicated in fibrosis [39], was activated in the eutopic and ectopic endometrium, and that the protective effects of MOR106 involve attenuation of fibrosis through inhibition of this signaling pathway.

### Neutralization of IL-17 C reduces M2-like macrophage infiltration and reduces inflammation

**Fig. 4:**
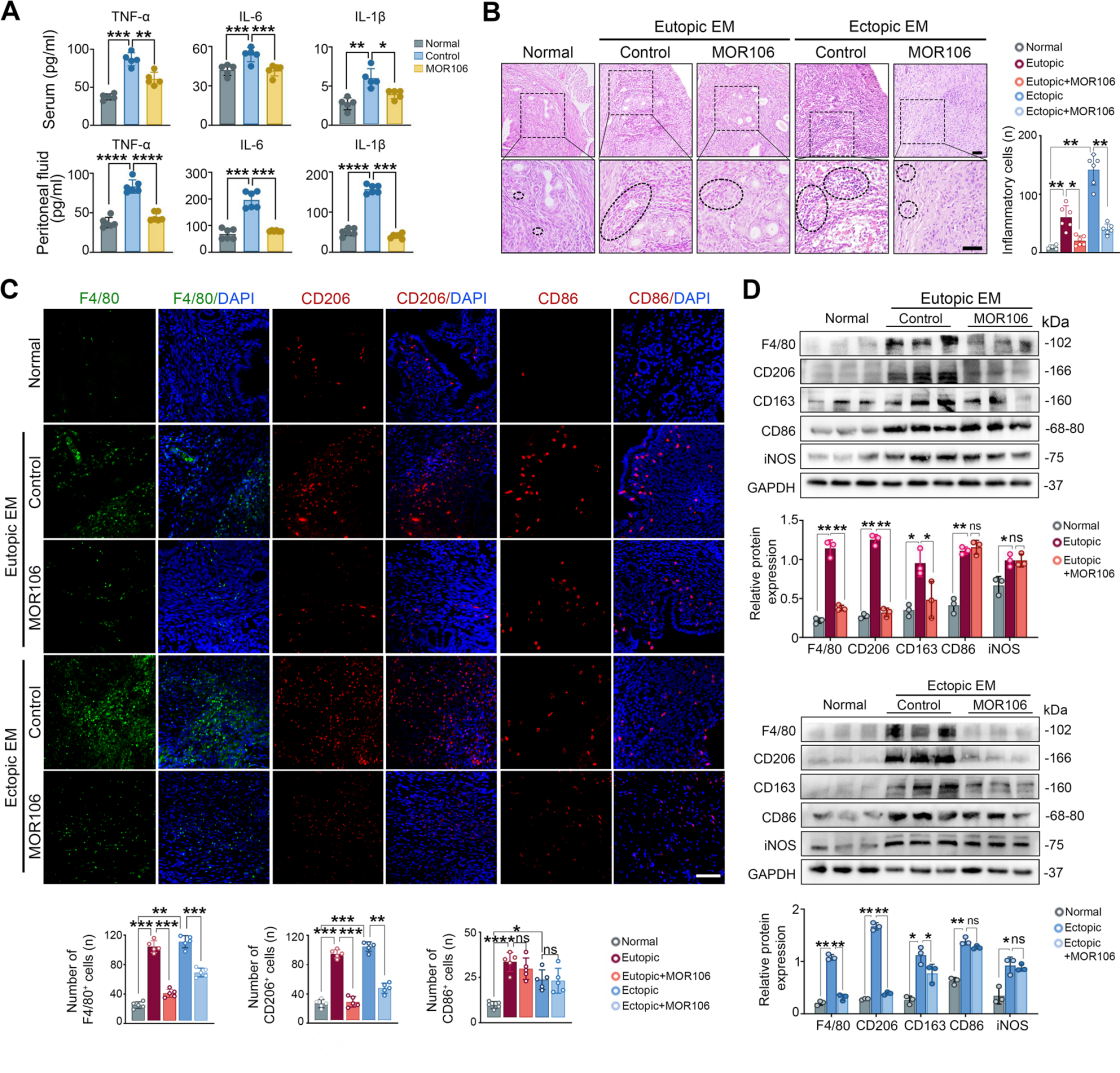
MOR106 attenuates inflammation and modulates macrophage polarization in endometriosis. (**A**) Quantification of inflammatory cytokines in peritoneal fluid and serum of EM mice. (**B**) The H&E staining showing inflammatory cells. (**C**) Immunofluorescence staining and (**D**) western blot analysis for macrophage markers in EM mice. Scale bars: 50 μm. ns, not significant. *P < 0.05, **P < 0.01 and ***P < 0.001 for the indicated comparisons. EM, endometriosis

Analysis of serum and peritoneal fluid demonstrated significantly elevated levels of key inflammatory cytokines TNF-α, IL-6, and IL-1β in the untreated EM group relative to controls. MOR106 treatment significantly reduced these elevated cytokine concentrations (Fig. 4A). Consistent with this reduced systemic inflammation, H&E staining demonstrated that MOR106 administration effectively attenuated inflammation in both eutopic and ectopic endometrial tissue in mice compared to the untreated EM controls (Fig. 4B). To further investigate macrophage infiltration and phenotype directly within the endometrial tissues, we performed immunofluorescence and western blot analyses. These demonstrated that MOR106 treatment reduced the infiltration of CD206^+^ and CD163^+^ M2-like macrophages while there was no significant change in the infiltration of CD86^+^ and iNOS M1-like macrophages in both eutopic and ectopic endometrium of the EM group (Fig. 4C and D, Supplementary Figure S11). These data suggest that IL-17 C neutralization with MOR106 attenuates the infiltration and/or polarization of CD206^+^ M2-like macrophages within the ectopic lesions. This reduction in M2-like macrophages likely contributes to the inhibition of lesion progression, consistent with the known pro-fibrotic/pro-angiogenic role of M2-like macrophages in endometriosis pathogenesis [23].

### M2-like macrophages promote stromal fibrosis via the MAPK/ERK pathway

**Fig. 5:**
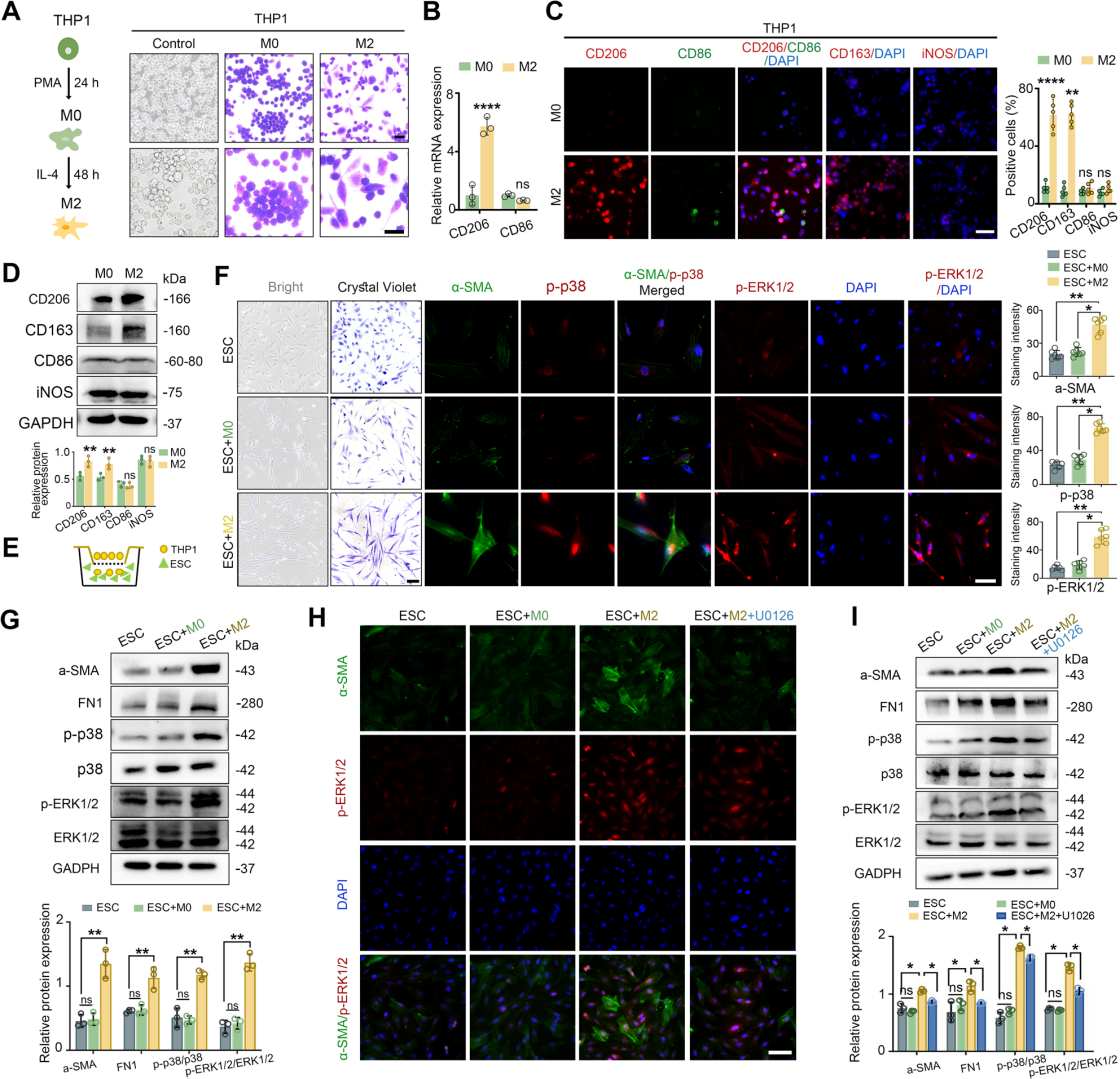
M2-like macrophages promote stromal fibrosis via MAPK/ERK signaling. (**A**) Schematic diagram of M0 and M2 polarization and morphological changes in THP-1-derived M0 and M2-like macrophages. (**B**) qRT-PCR, (**C**) immunofluorescence and (**D**) western blot analysis of CD206, CD86, CD163 and iNOS expression of THP-1 cells before and after polarization. (**E**) Schematic of THP-1-ESC co-culture. (**F**) ESC morphology and immunofluorescence co-localization of α-SMA, p-ERK1/2, and p-p38MAPK in ESCs under indicated conditions. (**G**) Western blot analysis of ECM-related and MAPK pathway key proteins in ESCs after co-culture. (**H**) Cells were treated with ERK inhibitor (U0126) under THP-1-ESC co-culture. Immunofluorescence co-localization of α-SMA and p-ERK1/2 in ESCs under indicated conditions. (**I) **The expression of ECM-related and MAPK pathway key proteins was detected by western blot. Scale bars: 50 μm. ns, not significant. *P < 0.05, **P < 0.01 and ****P < 0.0001 for the indicated comparisons

THP-1 cells were differentiated into M0 macrophages using PMA. The cells exhibited an observable oval-shaped appearance, with some fibroblast-like pseudopodia extending from the periphery, and began to adhere to a surface [40]. Upon subsequent induction with IL-4, the M2-like macrophages exhibited an adherent growth pattern, increased size, extended pseudopodia and a spindle-shaped morphology (Fig. 5A). Consistent with M2-like polarization, qRT-PCR, immunofluorescence assays and western blot analysis demonstrated that PMA/IL-4 treatment significantly increased CD206 and CD163 expression. Conversely, CD86 and iNOS expression levels showed no statistically significant change (Fig. 5B-D). Given the established role of fibroblast activation and ECM deposition in endometriosis fibrosis [8], we next investigated fibroblast-macrophage crosstalk using a 0.4 μm Transwell co-culture system. THP-1 cell-derived M2-like macrophages were seeded in the upper chamber, with primary ESCs in the lower chamber (Fig. 5E). After 24 h of co-culture with M2-like macrophages, ESCs underwent distinct morphological changes, adopting a spindle-shaped appearance similar to activated fibroblasts (Fig. 5F). Immunofluorescence colocalization staining further revealed increased expression of α-SMA, p-ERK1/2 and p−p38MAPK in these co-cultured ESCs (Fig. 5F). Western blot analysis of ESCs confirmed that co-culture with M2-like macrophages significantly upregulated the expression of fibrotic markers (FN1 and α-SMA) and activated the MAPK/ERK pathway, as evidenced by elevated levels of p-p38MAPK and p-ERK1/2 compared to control ESCs cultured alone (Fig. 5G). To functionally validate the involvement of the MAPK pathway, the ERK inhibitor U126 (10 µM) or p38MAPK inhibitor SB203580 (10 µM) was added to the M2-like macrophage-ESC co-culture system. Treatment with U126 or SB203580 significantly attenuated the M2 macrophage-induced upregulation of FN1, α-SMA, p-p38MAPK, and p-ERK1/2 in ESCs (Fig. 5H and I, and Supplementary Figure S12). These findings indicate that M2 macrophages promote fibrosis-associated changes in ESCs primarily through the activation of the MAPK signaling pathway specifically involving p38 and ERK.

### IL-17 C induces M2-like polarization and subsequent pro-fibrotic effects

**Fig. 6:**
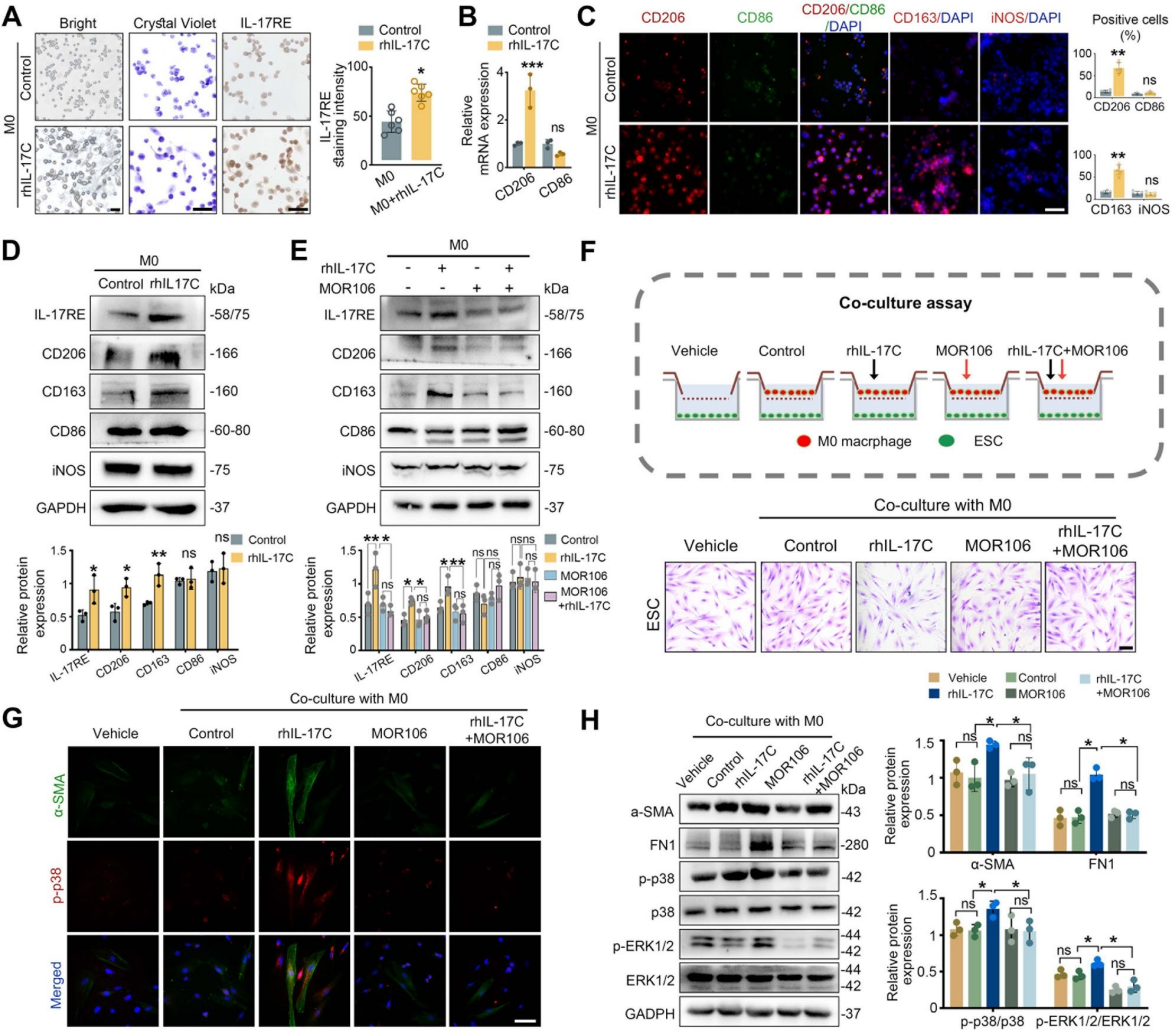
IL-17 C induces M2-like macrophage polarization and promotes fibrotic effects in ESCs. (**A**-**C**) M0 macrophages were treated with 100 ng/ml IL-17 C. The expression of IL-17RE and macrophage markers was detected by immunochemical staining, qRT-PCR, and (**D**) western blot. (**E**) M0 macrophages were treated with 100 ng/ml IL-17 C with or without MOR106 1 µg/ml. The expression of IL-17RE and macrophage markers were detected by western blot. (**F**) Schematic of M0 macrophages-ESCs co-culture, and morphology of ESCs from the parallel cell co-culture or non co-cultures. (**G**) Immunofluorescent expression of co-localization of α-SMA and p-p38MAPK expression in ESCs after co-culture. (**H**) M0 macrophages were treated with 100 ng/ml IL-17 C with or without MOR106 1 µg/ml, ECM -related and MAPK/ERK pathway key proteins were detected by western blot in ESCs after co-culture. Scale bars: 50 μm. ns, not significant. *P < 0.05, **P < 0.01 and ***P < 0.001 for the indicated comparisons

To determine the role of IL-17 C in macrophage polarization, M0 macrophages were treated with IL-17 C in vitro. Crystal violet staining showed PMA-treated THP-1 cells underwent differentiation into M0 macrophages, a process accompanied by observable oval-shaped appearance and they began to adhere to a surface. Upon the induction of 100 ng/ml rhIL-17 C for 48 h, M0 macrophages maintained oval-shaped morphology, exhibited extended pseudopodia and tended to aggregate for growth (Fig. 6A). The qRT-PCR results revealed significantly higher levels of CD206 mRNA expression in rhIL-17 C-treated M0 macrophages compared to control (Fig. 6B). Immunochemical staining revealed that IL-17 C treatment significantly enhanced the percentage of CD206^+^ and CD163^+^ cells while it did not influence the percentage of CD86^+^ and iNOS^+^ cells (Fig. 6C). Western blot analysis confirmed that IL-17 C treatment markedly increased CD206 and CD163 protein levels, consistent with M2-like polarization, and significantly upregulated the expression of IL-17RE, as ascertained by immunochemical staining (Fig. 6A and D). Importantly, treatment with the IL-17 C neutralizing antibody MOR106 (1 µg/ml) abolished the IL-17 C-induced upregulation of both IL-17RE, CD206 and CD163 in M0 macrophages (Fig. 6E).

Next, we investigated whether IL-17 C-induced macrophages could modulate ESC fibrotic activity. ECM-related gene expression was assessed in ESCs under different conditions: co-cultured with untreated or IL-17 C-treated M0 macrophages. Crystal violet staining showed IL-17 C treatment resulted in spindle-shaped morphology in ESCs co-cultured with M0 macrophages (Fig. 6F). When IL-17 C-treated macrophages were co-cultured with ESCs, immunofluorescence staining revealed increased expression of α-SMA and p-p38MAPK in ESCs (Fig. 6G). Consistently, Western blot analysis showed elevated phosphorylation of p38MAPK and ERK1/2, along with increased expression of ECM-related proteins in ESCs (Figure. 6G and H). Notably, blockade of IL-17 C signaling using MOR106 during this co-culture attenuated these pro-fibrotic effects and reduced the levels of p-p38MAPK and p-ERK1/2 in ESCs (Fig. 6G and H). These results implicate IL-17 C-induced M2-like macrophage polarization contributes to the activation of MAPK signaling and promotes the fibrotic response of ESCs.

## Discussion

Endometriosis is recognized as an inflammatory and fibrotic disease [3, 26, 39, 41]. Cytokines are key mediators in the establishment and progression of endometriosis [9, 19, 22], with cytokine-macrophage crosstalk driving myofibroblast activation during endometriosis-associated fibrosis [26]. However, specific mediators initiating the fibrotic cascade remain incompletely characterized. Using spatial transcriptomics and cellular interaction analyses, we compared endometriotic lesions to normal endometrium, confirming their inflammatory and fibrotic phenotype. Notably, IL-17 C levels were significantly elevated in peritoneal fluid, eutopic endometrium, and ectopic lesions of both human patients and mouse models. In vitro experiments demonstrated that IL-17 C promotes macrophage polarization toward an M2-like phenotype, evidenced by an increased CD206^+^ M2/CD86^+^ M1 ratio. Neutralization of IL-17 C reduces CD206^+^ M2 macrophage infiltration in eutopic endometrium, and ectopic lesions in a mouse model. These IL-17 C-induced M2-polarized macrophages enhanced ECM production in ESCs via activation of the MAPK/ERK signaling pathway. The novelty of this study is the identification of an IL-17 C-centered macrophage-stromal pro-fibrotic axis and the IL-17 C/IL-17RE axis as a novel therapeutic target for fibrosis in endometriosis.

The pathogenesis of endometriosis involves the retrograde endometrial shedding modulated by peritoneal immunity [42, 43]. Within this context, peritoneal fluid IL-17 has been implicated in the pathogenesis of endometriosis by inducing the production of cytokines such as IL-1β, IL-6 and IL-8 [44]. These pro-inflammatory cytokines are also downstream targets of IL-17 C signaling [45]. In particular, IL-17 C expression precedes that of IL-17 A during disease progression [46]. Previous spatial transcriptomics indicates minimal alterations in the sub-epithelial stroma but increased immune response in the endometriotic lesion compared to the matched eutopic epithelium [22]. Similarly, the markedly elevated concentrations of IL-17 C were found in the peritoneal fluid, eutopic endometrium, and ectopic lesions of patients with endometriosis compared to controls. The IL-17 C/IL-17RE axis is established in chronic inflammation and autoimmunity [38, 47], consistent with our transcriptomic data showing upregulation of this pathway in endometriosis-associated macrophages.

Emerging evidence reveals the pivotal role of macrophage polarization and the associated fibrotic response in the pathogenesis of endometriosis [20, 26]. Macrophage polarization is a dynamic and heterogeneous process, and the conventional classification of macrophages into M1-like and M2-like phenotypes represents an oversimplified framework. An M2-polarized phenotype mediates the process of immunosuppression, a hallmark of disease progression, and can promote lesion growth and fibrotic remodeling in endometriosis [48]. Both peritoneal fluid macrophages and macrophages infiltrating ectopic lesions exhibit features consistent with an M2-like phenotype [23, 42]. Importantly, experimental depletion of CD206^+^ M2-like macrophages significantly attenuates the formation of endometriosis-like lesions in mouse models [49]. Recent single-cell transcriptomic studies have further demonstrated that tissue macrophages, including those residing in inflammatory and fibrotic microenvironments, comprise heterogeneous populations with overlapping and dynamic transcriptional states rather than discrete M1 or M2 subsets [27, 28]. In endometriotic lesions, macrophages may exhibit mixed inflammatory, angiogenic, immunoregulatory, and pro-fibrotic signatures, depending on local cytokines profiles, hypoxia, estrogenic stimulation, and interactions with stromal and epithelial cells [8, 34]. In this study, CD86, iNOS, CD206 and CD163 were used as representative markers to assess macrophage polarization. However, additional M2-associated markers, such as IL-10, TGF-β, CCL18, as well as molecules involved in ECM remodeling, would provide a more comprehensive characterization of macrophage activation states. Therefore, the IL-17 C-induced macrophage phenotype observed in our study should be interpreted as an M2-like pro-fibrotic activation state rather than a fixed M2 lineage.

Multiple studies have explored the role of IL-17 A in driving fibrosis in lung and kidney; however, the function of IL-17 C in fibrotic diseases remains less well characterized [50]. Some studies show IL-17 C may amplify inflammatory responses by activating and recruiting macrophages expressing IL-17RE, thereby contributing to fibrotic remodeling [45, 51]. IL-17 C blockade could reduce ECM accumulation and macrophage infiltration in kidney and lung fibrosis models [45, 50]. Nevertheless, the reported effects of IL-17 family cytokines on macrophage polarization remain controversial. Some studies indicate that IL-17 signaling increases the M1/M2 macrophage ratio [51, 52], implying that its function may be highly dependent on the tissue microenvironment. In addition, IL-17 A can directly induce M2-like macrophage polarization to promote lesion development in endometriosis [53].

In our study, ST analysis revealed that IL-17 C-associated regions were not randomly distributed within ectopic lesions, but were closely linked to specific local microenvironmental features, including inflammatory infiltration, macrophage-enriched signals, and ECM remodeling. In particular, IL-17 C-high regions were spatially adjacent to fibrotic stromal compartments characterized by elevated expression of ECM-related genes. These findings suggest that IL-17 C signaling may be preferentially activated within inflammatory-fibrotic niches of endometriotic lesions. Therefore, we propose that ectopic lesions may ‘hijack’ IL-17 C signaling to sustain a pathological repair program: Chronic inflammatory stimulation and tissue injury may induce epithelial or stromal IL-17 C production, while the surrounding ECM-rich microenvironment facilitates macrophage recruitment, profibrotic polarization, and fibroblast activation. Through this spatially organized macrophage-fibroblast interaction, IL-17 C may contribute to persistent inflammation, ECM deposition, and fibrotic lesion progression.

Importantly, IL-17 C expression was more directly associated with fibrotic features, including increased expression of ECM markers. These findings support the possibility that IL-17 C is particularly relevant to the fibrotic remodeling process in endometriosis. Specifically, IL-17 C may promote macrophage phenotypes associated with ECM deposition, thereby amplifying fibrosis in endometriotic lesions. Consistent with previous reports suggesting context-dependent effects of IL-17 signaling, our results confirmed that IL-17 C was associated with an increased M1/M2 macrophage ratio in the eutopic endometrium, whereas it promoted M2-like macrophage polarization in ectopic lesions and in human macrophages in vitro. These findings indicate that the effect of IL-17 C on macrophage polarization depends on the local environment. Our current findings establish a direct link between IL-17 C and M2-like macrophage polarization. This IL-17 C-M2 macrophage axis promotes macrophage infiltration, inflammation and fibrosis in animal models of endometriosis. Moreover, targeting this axis effectively ameliorates fibrosis in our experimental mice, suggesting that IL-17 C may represent a potential therapeutic target for fibrotic progression in endometriosis.

While the presence of ectopic endometrial glands and stroma represents the initiating pathological event and diagnostic hallmark, the fibrotic phenotype is increasingly recognized as central to endometriosis pathology, often persisting even when epithelial elements are absent and replaced by dense ECM [3, 7]. Myofibroblasts, derived primarily via fibroblast to myofibroblast transition (FMT), are the main secretors of ECM [26, 54]. Aberrant MAPK/ERK signaling promotes recruitment and differentiation of fibroblasts into myofibroblasts, thereby exacerbating fibrotic remodeling in endometriosis [55]. Binding sites for transcription factors, such as NF-kB and MAPK signaling molecules, have been identified in the promoter region of IL-17 C, contributing to the inflammatory responses and tissue destruction [50]. Moreover, IL-17 C signaling has been linked to fibrotic pathways by acting on fibroblasts in several other diseases [56–58]. Recent advances in intraperitoneal and local cytokine therapy, such as IL-8 and IL-33, have been explored and may be effective strategies for relieving fibrosis through their regulation of immune cell recruitment and fibroblast activation [59, 60]. Similarly, our findings demonstrated that IL-17RE was expressed and localized to fibroblasts within endometriosis, suggesting a direct activation by IL-17 C.

Pharmacological inhibition of MAPK/ERK attenuates lesion growth in endometriosis models and blocks FMT in lung and hepatic fibrosis, suggesting its central role in fibrogenesis [54, 61]. In our study, transcriptomic analyses of ectopic tissues demonstrated pronounced immune cell infiltration, transcriptional reprogramming linked to ECM remodeling and inflammation, and upregulation of multiple pro-fibrotic genes. In vitro assays demonstrate that MAPK/ERK activation not only initiates fibroblast transdifferentiation but also upregulates pro-fibrotic gene networks. Additionally, the IL-17 C/IL-17RE signaling cascade activates the MAPK pathway by phosphorylation of p38, ERK, or JNK (c-Jun N-terminal kinase) [46]. Our findings indicate that IL-17 C does not necessarily act directly on ESCs to activate MAPK/ERK signaling. Instead, IL-17 C stimulation promotes M2-like macrophage polarization, and these polarized macrophages subsequently enhance ERK/MAPK activation in co-cultured ESCs. This supports a macrophage-dependent mechanism in which IL-17 C contributes to fibrotic remodeling by modulating macrophage-stromal cell crosstalk within the endometriotic lesion microenvironment.

## Conclusions

In summary, our spatial transcriptomic atlas of endometriotic lesions and matched normal endometrium revealed cellular crosstalk and phenotypic transformation within lesions, with macrophages orchestrating fibroblast-to-myofibroblast transition. We identify IL-17 C as a pivotal mediator of fibrosis, driving both M2-like macrophage polarization and subsequent fibroblast activation through macrophage-derived signals and MAPK/ERK pathway stimulation. Inhibition of the IL-17 C-M2 axis thus represents a promising therapeutic strategy against endometriosis-associated fibrosis.

### Limitations of the study

This study has several limitations. First, this study is limited by the relatively small number of ST samples and the absence of matched single-cell RNA-seq references, which precluded the use of Robust Cell Type Decomposition (RCTD). Larger patient cohorts and expanded ST datasets will be needed to validate the reproducibility and clinical relevance of the IL-17 C-associated inflammatory–fibrotic niche.

Second, although our results suggest that IL-17 C is closely associated with fibrotic remodeling in endometriosis, the current data do not fully determine whether IL-17 C acts independently or synergistically with other IL-17 family members, particularly IL-17 A. IL-17 C may cooperate with IL-17 A or other IL-17 cytokines to amplify inflammation and fibrosis. Future studies are required to clarify the relative and cooperative contributions of IL-17 C within the broader IL-17 cytokine network.

Third, IL-17RE is expressed in both macrophages and fibroblasts, which prevents definitive assignment of its cell-type-specific function. Although our data suggest that IL-17 C promotes macrophage pro-fibrotic polarization via IL-17RE and indirectly activates fibroblasts through macrophage-mediated MAPK/ERK signaling, direct effects on fibroblasts cannot be excluded. Cell-type-specific IL-17RE knockout models will be necessary to validate its role in this pro-fibrotic macrophage-fibroblast circuit.

## Supplementary Information

Below is the link to the electronic supplementary material.

Supplementary Material 1

Supplementary Material 2

Supplementary Material 3

Supplementary Material 4

## Data Availability

The raw sequencing data in this study have been deposited in Gene Expression Omnibus (GSE291389) and are publicly available at https://www.ncbi.nlm.nih.gov/geo/query/acc.cgi?acc=GSE291389. Any additional information required to reanalyze the data reported in this paper is available from the lead contact upon request.

## Abbreviations

ST: Spatial transcriptomics
EM: Endometriotic lesions
IL-17C: Interleukin-17C
IL-17RE: Interleukin-17 Receptor E
MOR106: Anti-IL-17C monoclonal antibody MOR106
M2: M2 Macrophage
MAPK: Mitogen-Activated Protein Kinase
ERK: Extracellular Signal-Regulated Kinase
ESCs: Endometrial stromal cells
ECM: Extracellular matrix
UMAP: Uniform Manifold Approximation and Projection
OCT: Optimal Cutting Temperature
KEGG: Kyoto Encyclopedia of Genes and Genomes
GO: Gene Ontology
GSVA: Gene Set Variation Analysis
ELISA: Enzyme-linked immunosorbent assay
IHC: Immunohistochemistry
IF: Immunofluorescence
FN1: Fibronectin 1
α-SMA: α-smooth muscle actin
