# Supplementary material for: Targeting the IL-17C-M2 macrophage axis ameliorates fibrosis in endometriosis through MAPK/ERK signaling
Source: J Transl Med. 2026 Aug 5;24:1015. doi: 10.1186/s12967-026-08769-9 (PMC13452111; doi:10.1186/s12967-026-08769-9)
Supplement: Supplementary file 1 [file 12967_2026_8769_MOESM1_ESM.pdf]

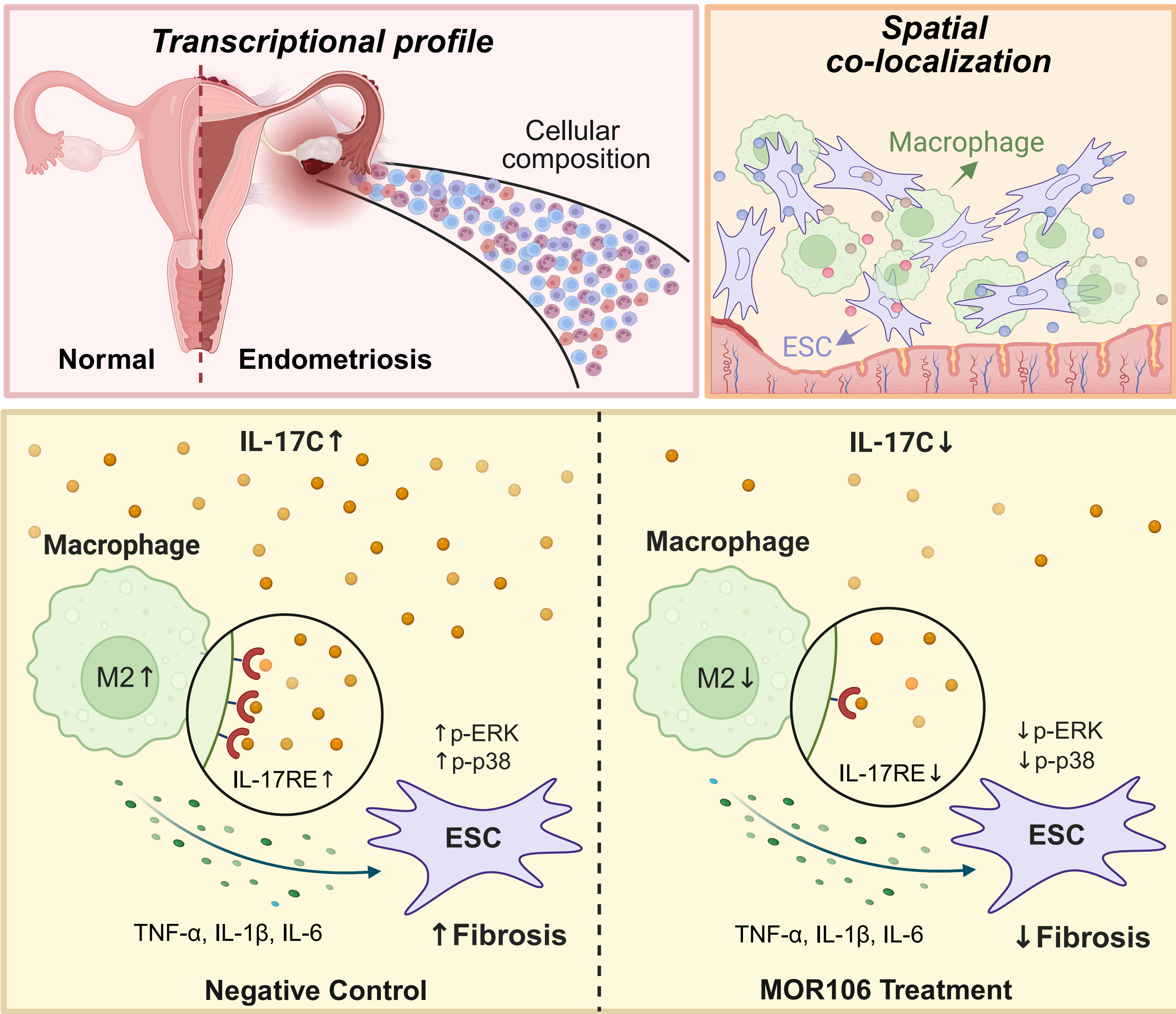

Transcriptional profile
Cellular composition
Normal
Endometriosis
Spatial co-localization
Macrophage
ESC
IL-17C↑
Macrophage
M2↑
IL-17RE↑
↑p-ERK
↑p-p38
ESC
TNF-α, IL-1β, IL-6
↑Fibrosis
Negative Control
IL-17C↓
Macrophage
M2↓
IL-17RE↓
↓p-ERK
↓p-p38
ESC
TNF-α, IL-1β, IL-6
↓Fibrosis
MOR106 Treatment
