# Supplementary material for: Targeting the IL-17C-M2 macrophage axis ameliorates fibrosis in endometriosis through MAPK/ERK signaling
Source: J Transl Med. 2026 Aug 5;24:1015. doi: 10.1186/s12967-026-08769-9 (PMC13452111; doi:10.1186/s12967-026-08769-9)

Full unedited gel for figure 3.

E

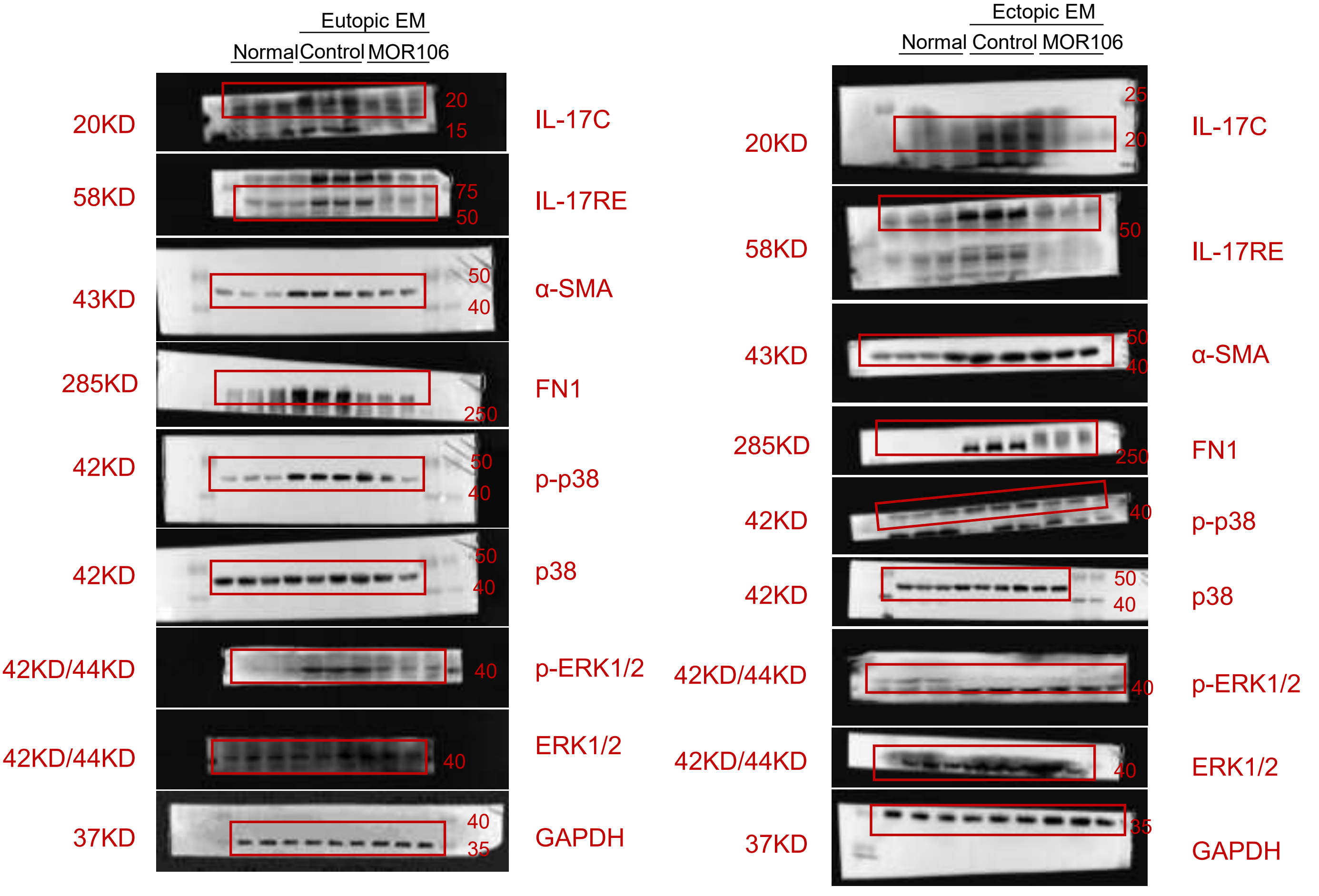

Full unedited gel for figure 4.

E

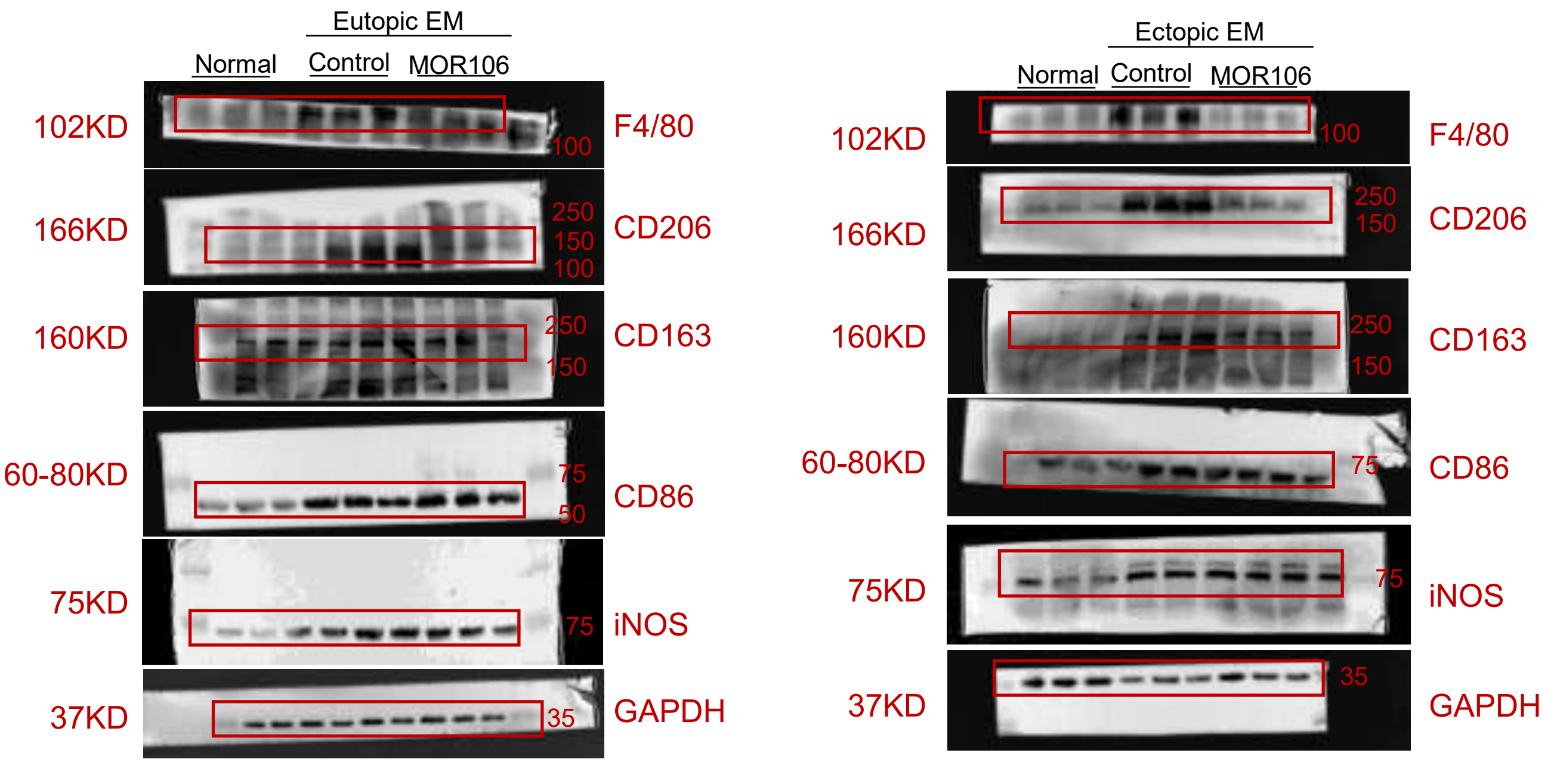

Full unedited gel for figure 5.

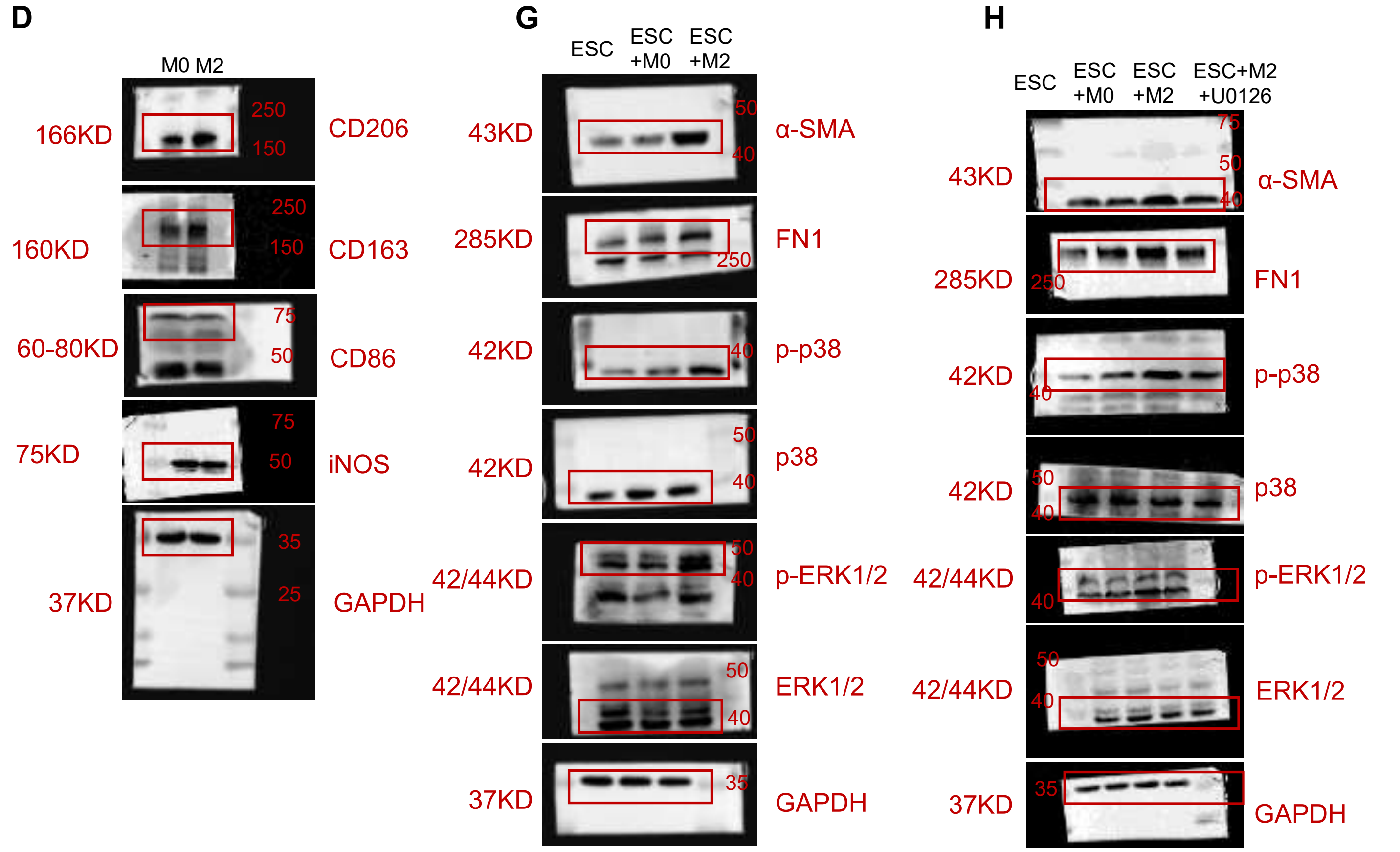

Full unedited gel for figure 6.

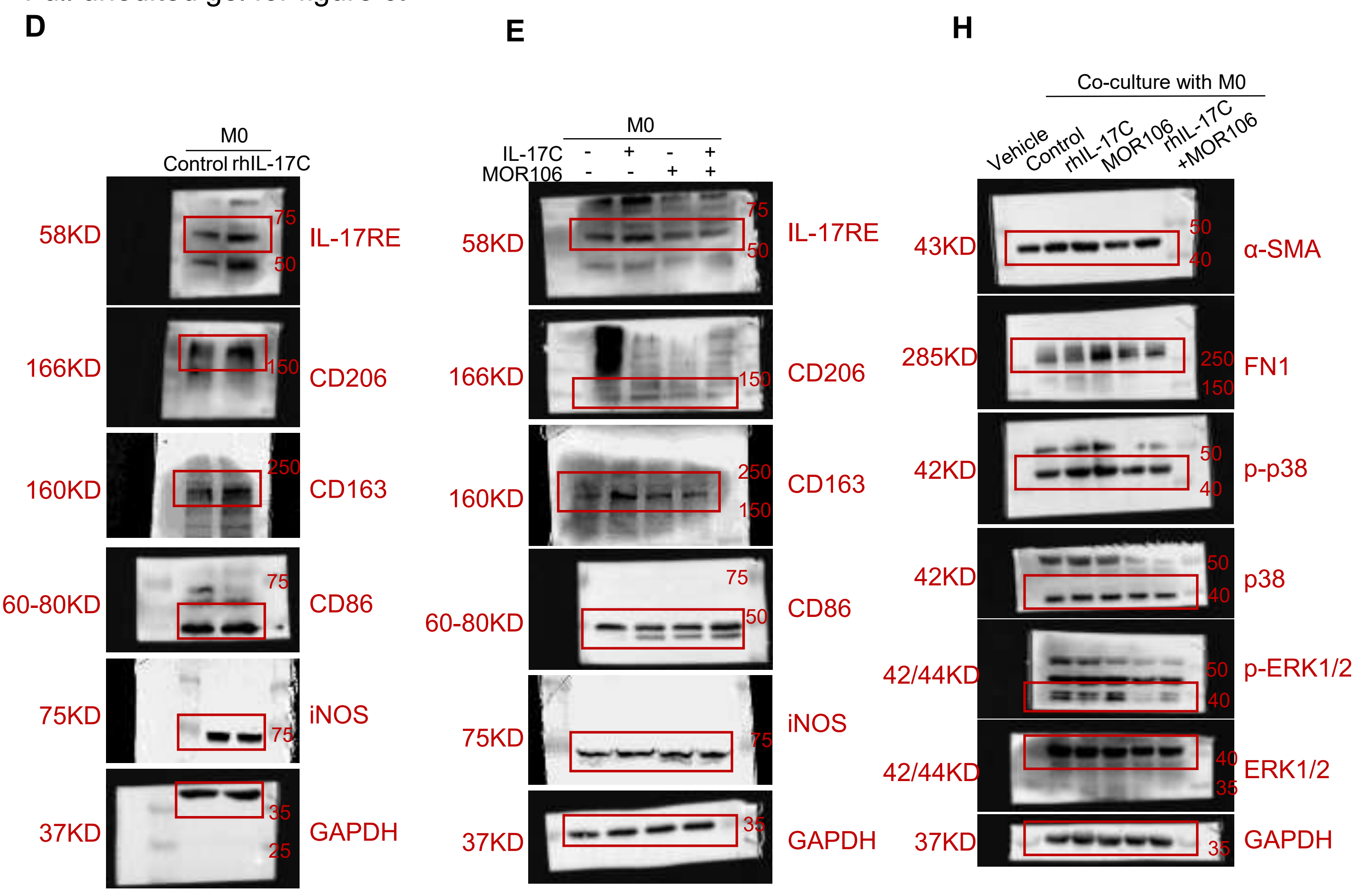

Full unedited gel for figure S12.

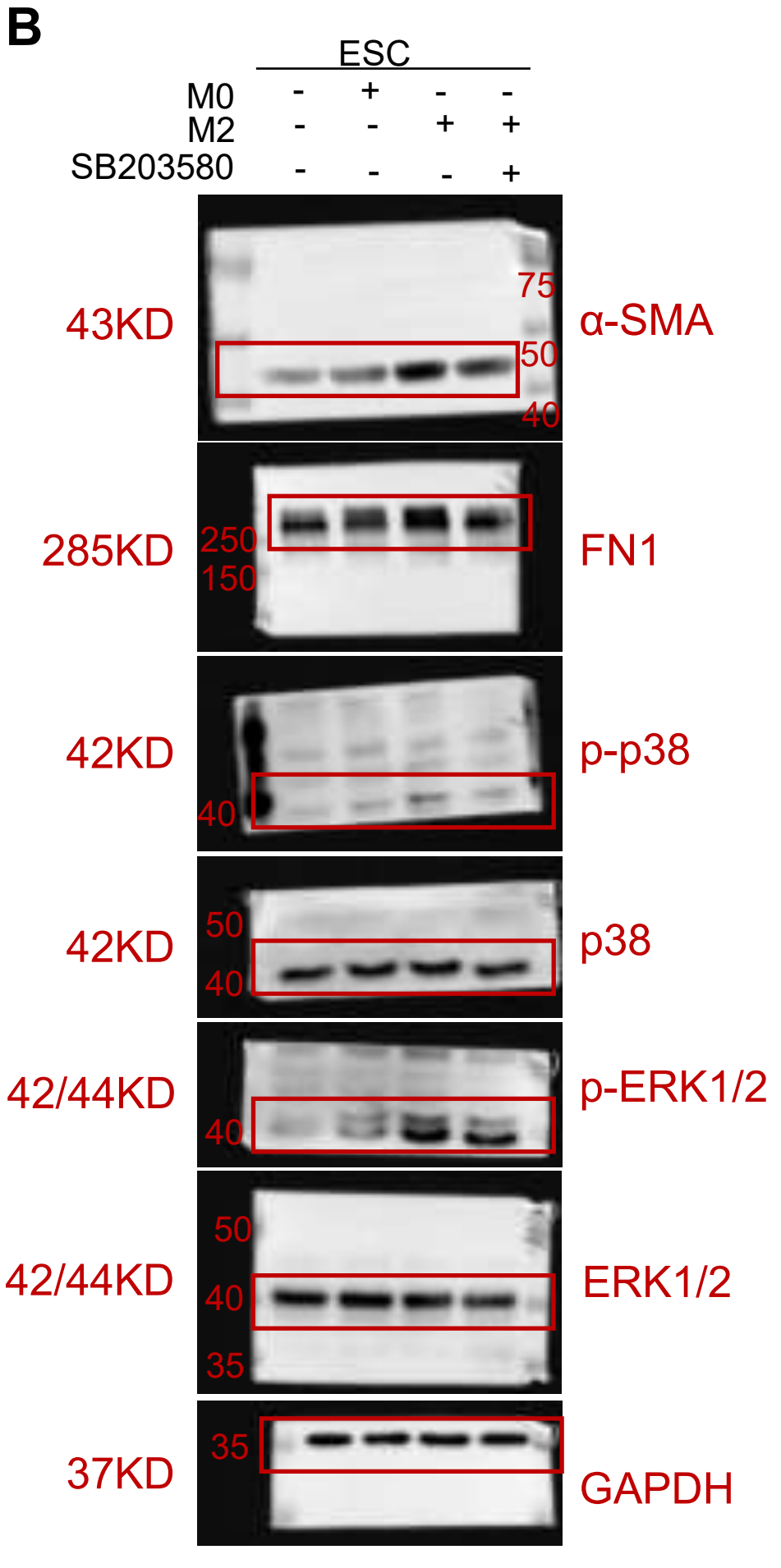

Supplement: Supplementary file 2 [file 12967_2026_8769_MOESM2_ESM.pdf]
