# Supplementary material for: Targeting the IL-17C-M2 macrophage axis ameliorates fibrosis in endometriosis through MAPK/ERK signaling
Source: J Transl Med. 2026 Aug 5;24:1015. doi: 10.1186/s12967-026-08769-9 (PMC13452111; doi:10.1186/s12967-026-08769-9)
Supplement: Supplementary file 3 [file 12967_2026_8769_MOESM3_ESM.pdf]

Supplementary Tables S1. Demographic and pathological data of the two samples for spatial transcriptomics (ST) analysis.

Case	Sample type	Age	Gravidity /Parity	r-AFS stage	CA125 (IU/ml)	Diameter (cm)	Pelvic Fluid （ml）
1	Normal	30	0	-	-	-	-
2	Endometriomas(EM)	40	G3P2	Ⅱ	330	Cyst: 41×29 ×58 mm	21

EM, endometriosis; Gravidity, G; Parity, P; r-AFS: revised American Fertility Society.

Supplementary Tables S2. Clinical characteristics of the patients for histology, gene expression and ELISA analysis.

	Normal	Endometriosis
Number of cases (n)	25	28
Age (years)	38.4±3.7	40.0±6.4
Type		
Ovarian endometriosis	–	15
Abdominal wall endometriosis	–	13

Supplementary Tables S3. Marker genes used for annotation

Subtype	Marker
Endometrial Gland	EPCAM, CDH1
Fibrotic stroma	ACTA2, FN1, COL1A1
Immune cell	CD79A, CSF3R, IL2RB, GZMM
Endometrial Stroma	COL6A1, VIM
Ovarian Stroma	SVIL, PRELP
Blood Vessel	MCAM, CLDN5
Blood Area	PLAUR, TXLNA
CD206+ macrophage	CD68, CD14, CD163, MRC1
CD86+ macrophage	CD68, CD14, CD163, CD86
Fibrotic stroma-1	VIM, COL6A1
Fibrotic stroma-2	ACTA2, COL6A1
T-B cell	CD79B, MZB1,CD79A, CD3E,CD3D,TRAC
Smooth muscle cell	RGS5, MCAM, PDGFRB
Endothelial cell	PECAM1, CD34, ESAM, CLDN5

Supplementary Tables S4. Antibodies used for immunostaining, immunofluorescence and western blot.

Name	Brand
CD86	proteintech
CD206	proteintech
F4/80	proteintech
CD163	Cell Signaling Technology
iNOS	proteintech
ERK1/2	Affinity Biosciences
Phospho-ERK1/2	Affinity Biosciences
p38 MAPK	Cell Signaling Technology
Phospho-p38 MAPK (Thr180/Tyr182)	Cell Signaling Technology
Fibronectin	Abcam
α-SMA	Cell Signaling Technology
IL-17C	Bioss
IL-17RE	proteintech
GAPDH Mouse Monoclonal Antibody	Cell Signaling Technology

Supplementary Tables S5. Specific primers.

Primer name	Primer sequence（5’-3’）
CD86-F	CCTTCCTGCTCTCTGCTAACTT
CD86-R	TAGGTTCTGGGTAACCGTGT
CD206-F	GCCTCGTTGTTTTGCGTCTT
CD206-R	GAGAACAGCACCCGGAATGA
GAPDH-F	GGAGCGAGATCCCTCCAAAAT
GAPDH-R	GGCTGTTGTCATACTTCTCATGG
