# Supplementary material for: Targeting the IL-17C-M2 macrophage axis ameliorates fibrosis in endometriosis through MAPK/ERK signaling
Source: J Transl Med. 2026 Aug 5;24:1015. doi: 10.1186/s12967-026-08769-9 (PMC13452111; doi:10.1186/s12967-026-08769-9)
Supplement: Supplementary file 4 [file 12967_2026_8769_MOESM4_ESM.pdf]

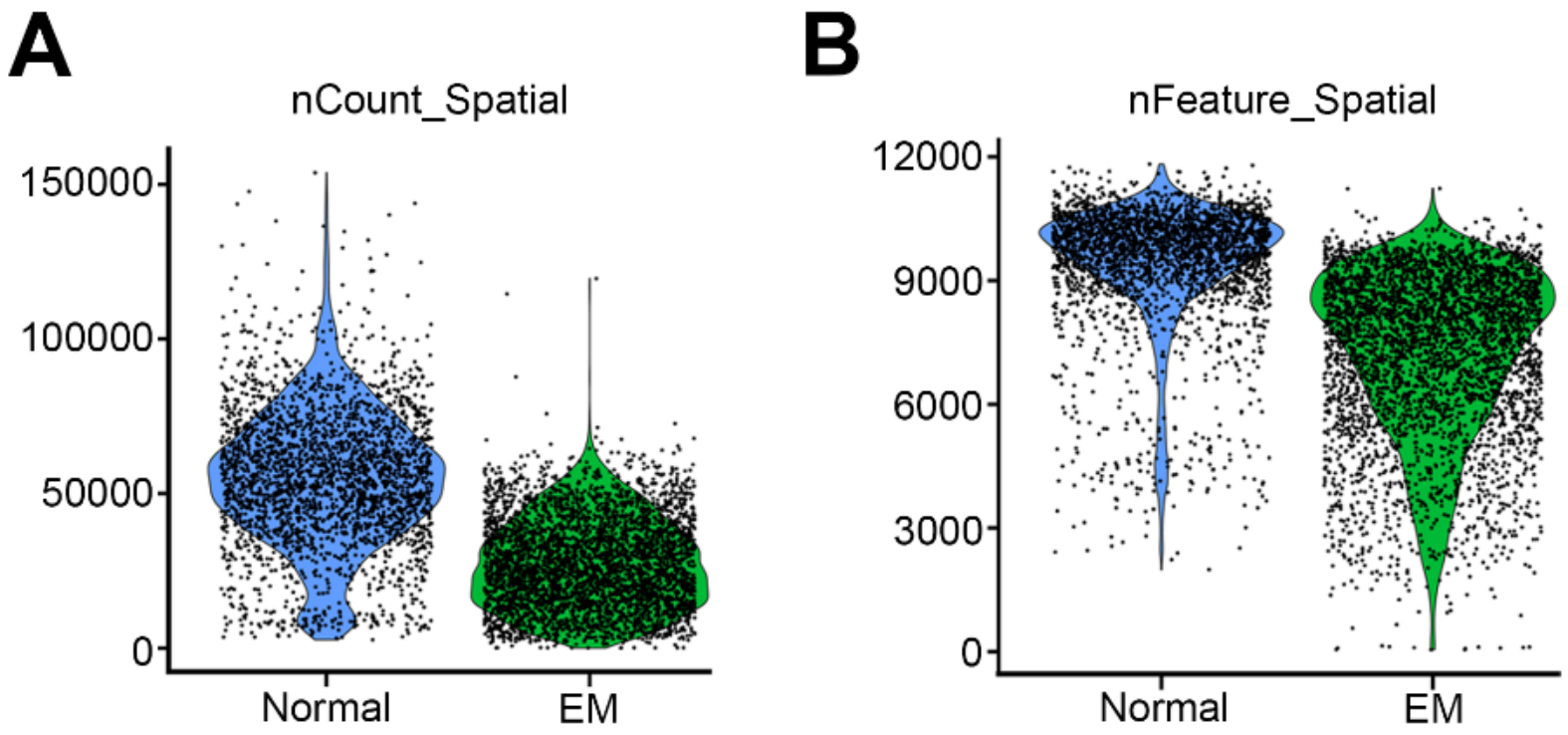

Supplementary Figure S1. Quality of the ST atlas. (A) The ST data were for 2648–3848 spots per tissue, and each spot contained approximately 50-153761 unique molecular identifiers (UMIs) and (B) 50-11822 genes. nFeature, number of features. EM, endometriosis.

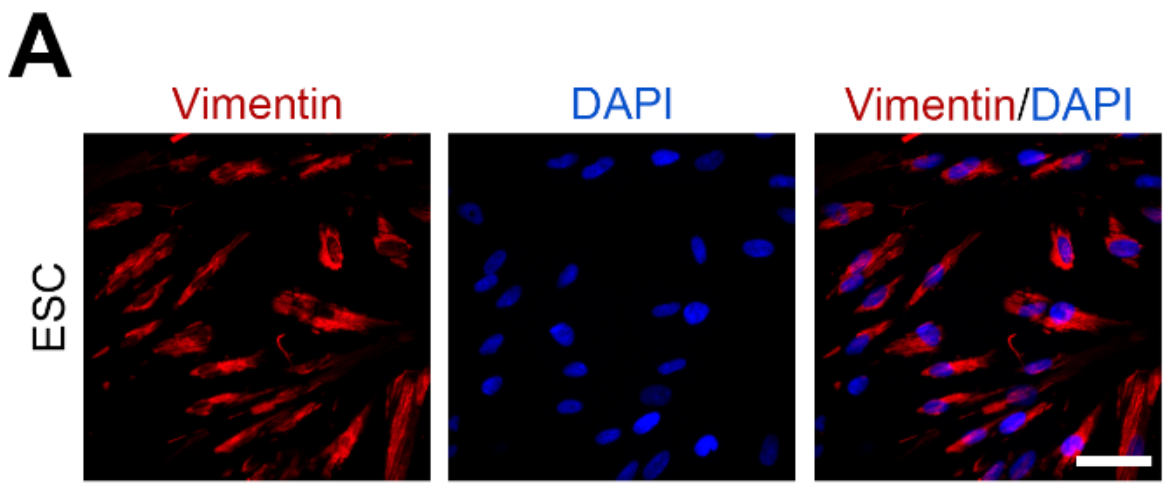

Supplementary Figure S2. Primary endometrial stromal cells identification. (A) Isolated stromal cells were stained with stromal cell marker vimentin. Scale bars: 50 μm.

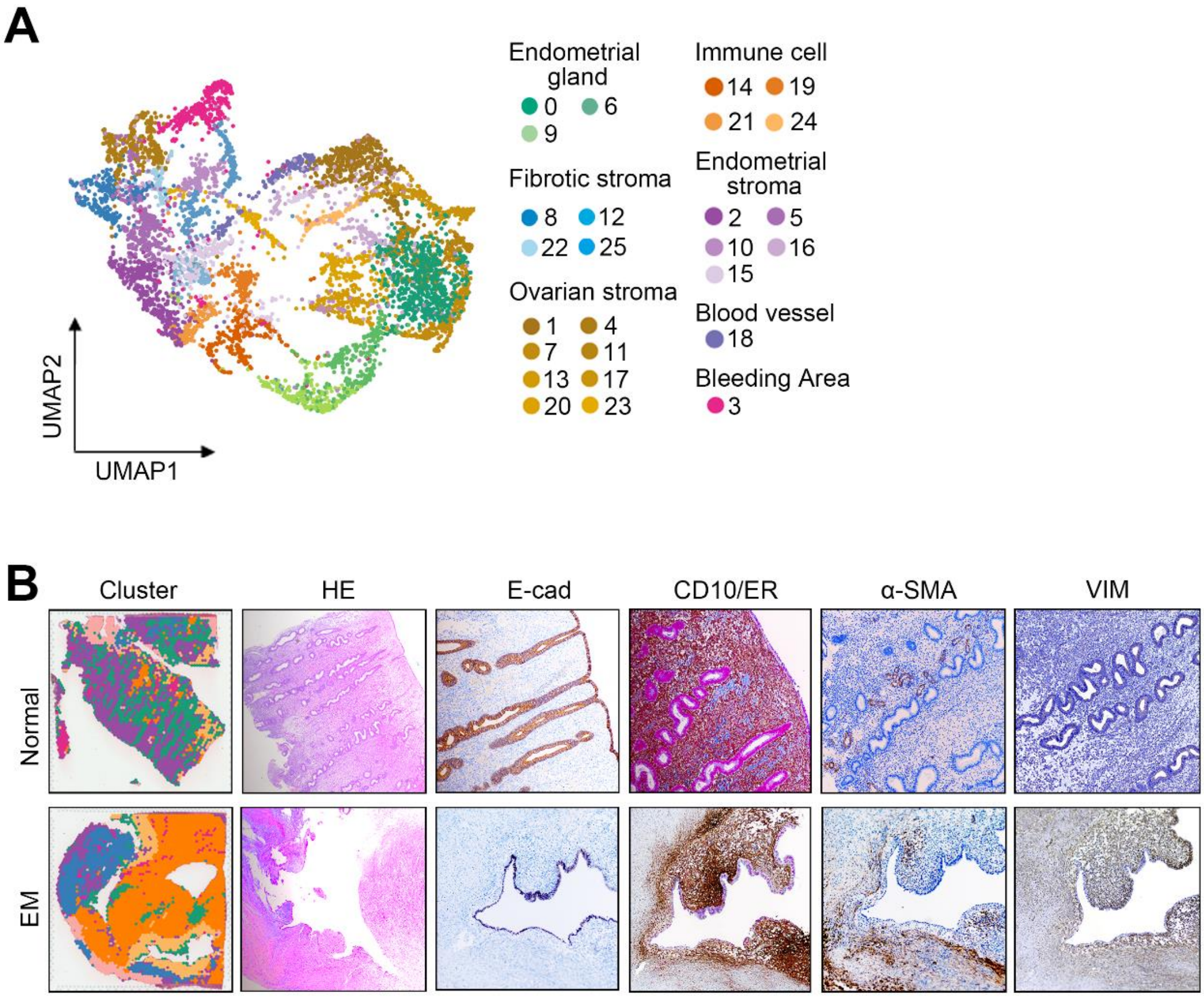

Supplementary Figure S3. Spatial transcriptomics atlas of normal and endometrioma (EM). (A) Uniform manifold approximation and projection plot of all spots from the two tissues. Twenty-six clusters were identified. (B) Prediction of the cell-type profiles of the spatial transcriptomics niches.

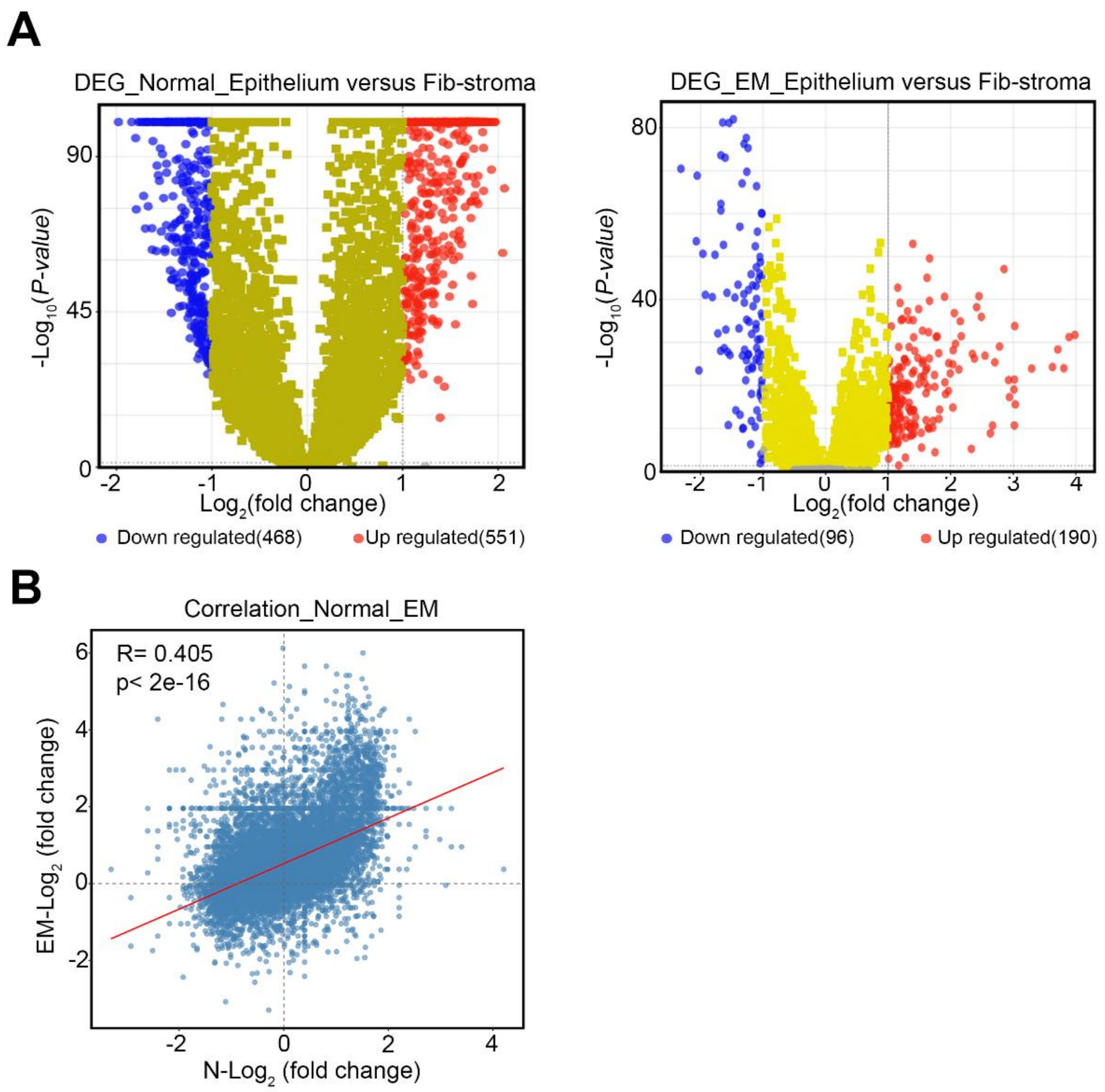

Supplementary Figure S4. Epithelium versus Stroma in endometriotic lesion and control endometrium. (A) Volcano plots highlighting differentially expressed genes (DEG) in the endometriotic lesion and control endometrium comparisons. There were 286 DEG (190 increased and 96 decreased) in the lesions and 1372 DEG in control endometrium (551 increased and 468 decreased). (B) Cell compartment gene expression was highly correlated in the endometriotic lesion and control endometrium.

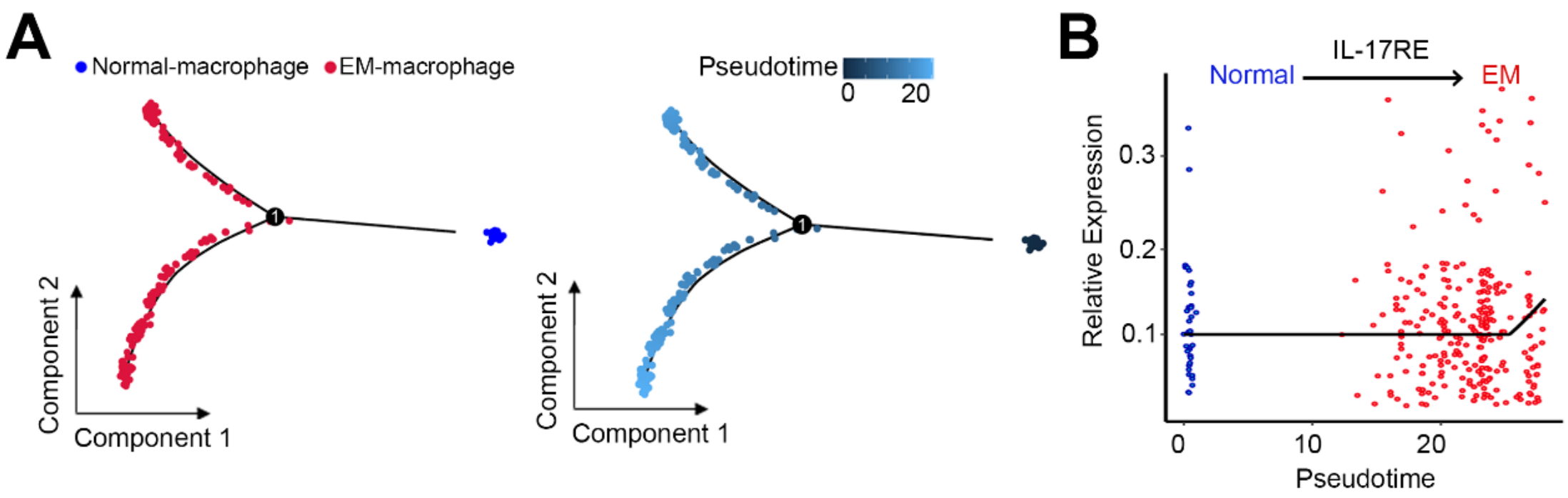

Supplementary Figure S5. Pseudotime trajectory analysis of macrophages inferred by Monocle v2. (A) Pseudotime trajectory of macrophage populations. (B) Dynamic expression pattern of IL17RE along the macrophage pseudotime trajectory.

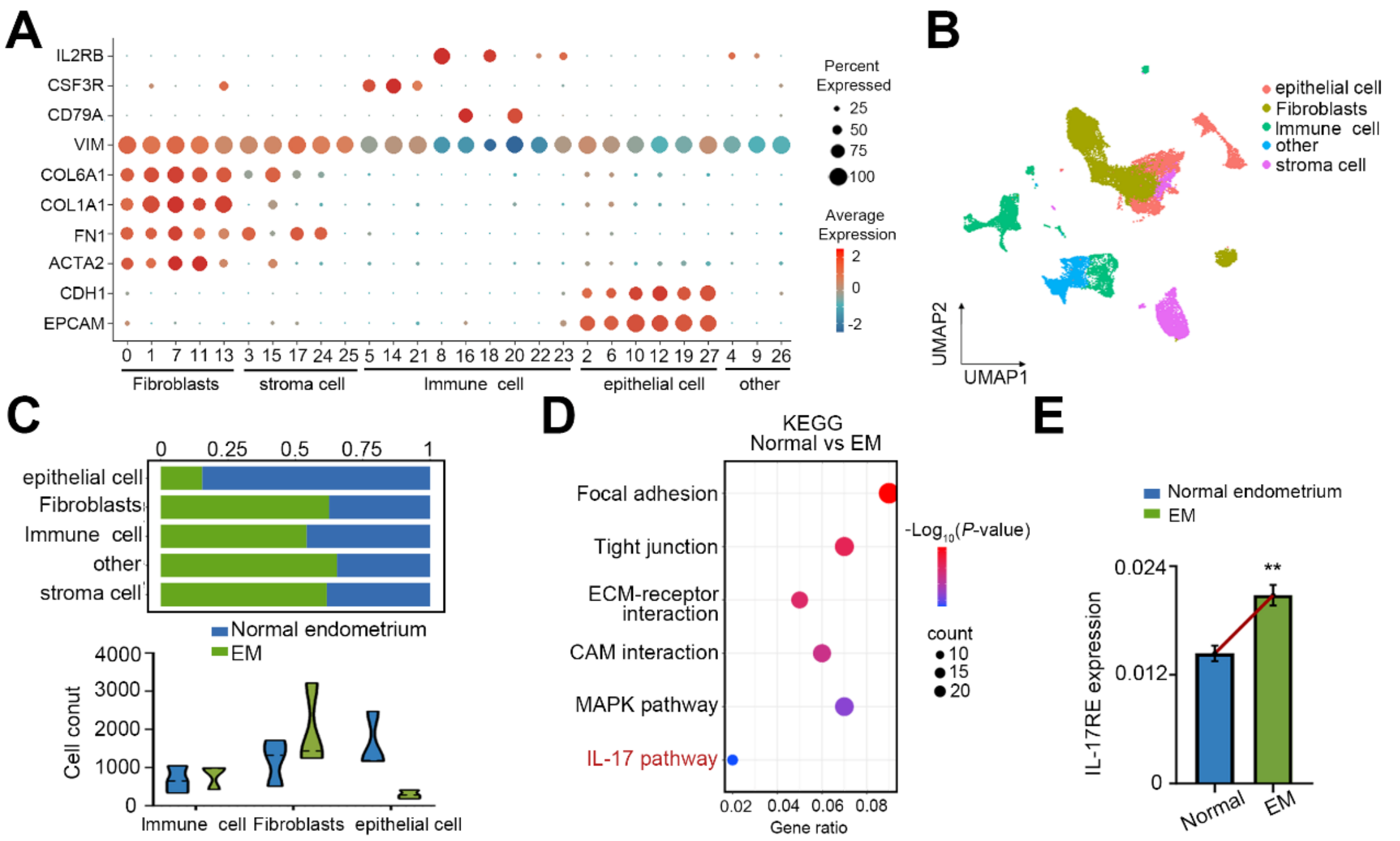

Supplementary Figure S6. External validation of findings using the GSE179640 dataset. (A) Dot plot of canonical marker genes identifying major cell lineages in ovarian EM and normal endometrial tissues. (B) UMAP plot showing the distribution of identified cell populations. (C) Comparison of cell type proportions and abundance between groups. (D) KEGG enrichment analysis highlighting activated pathways in EM lesions. (E) Expression levels of IL-17RE in ovarian EM lesions versus control. ***P* < 0.01.

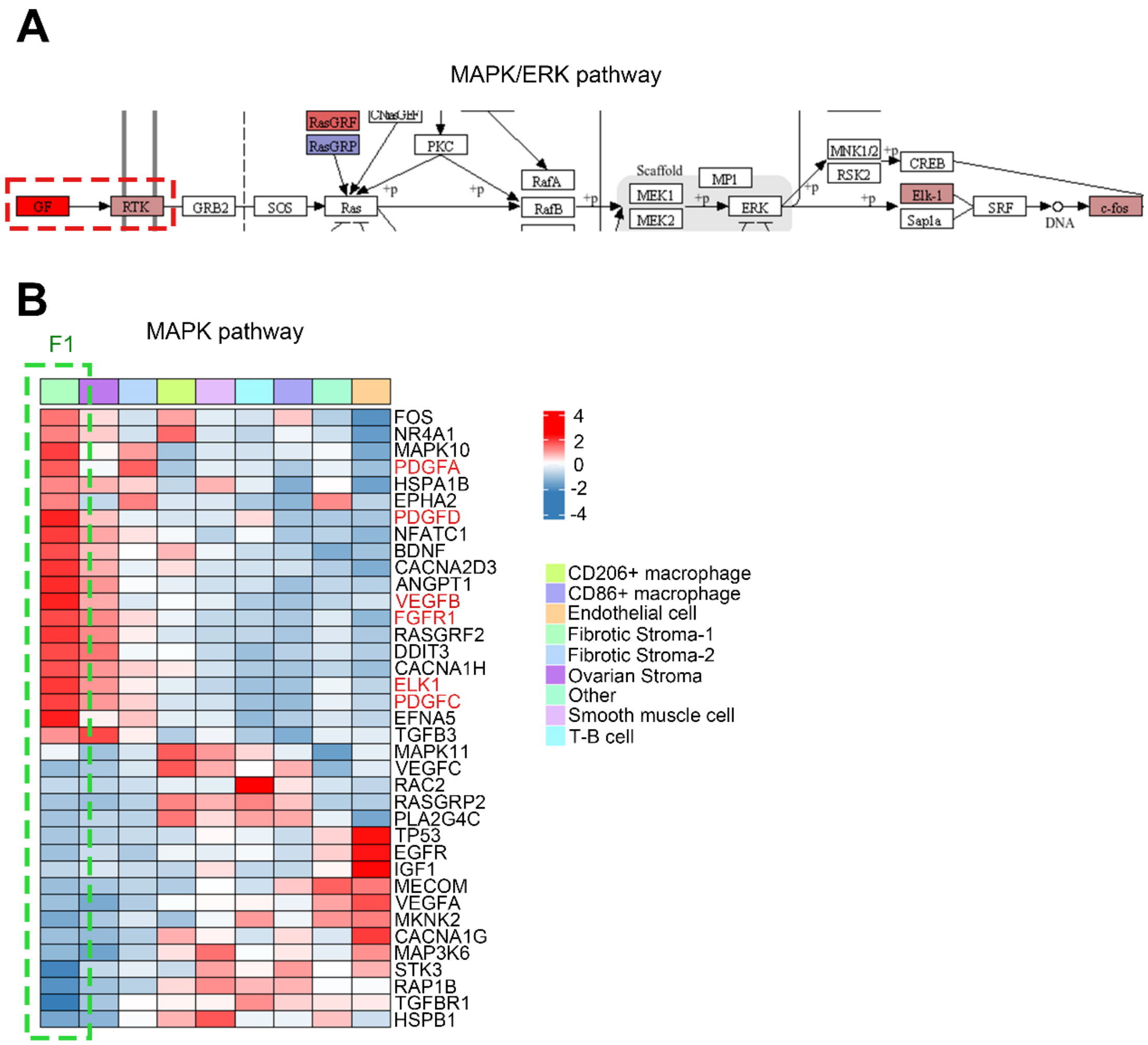

Supplementary Figure S7. The MAPK/ERK signaling pathway was hyper-activated in cluster of fibrotic stroma. (A) The KEGG map of MAPK/ERK signaling pathway. (B) Differentially expressed genes of Fibrotic stroma-1 enriched in the MAPK pathway.

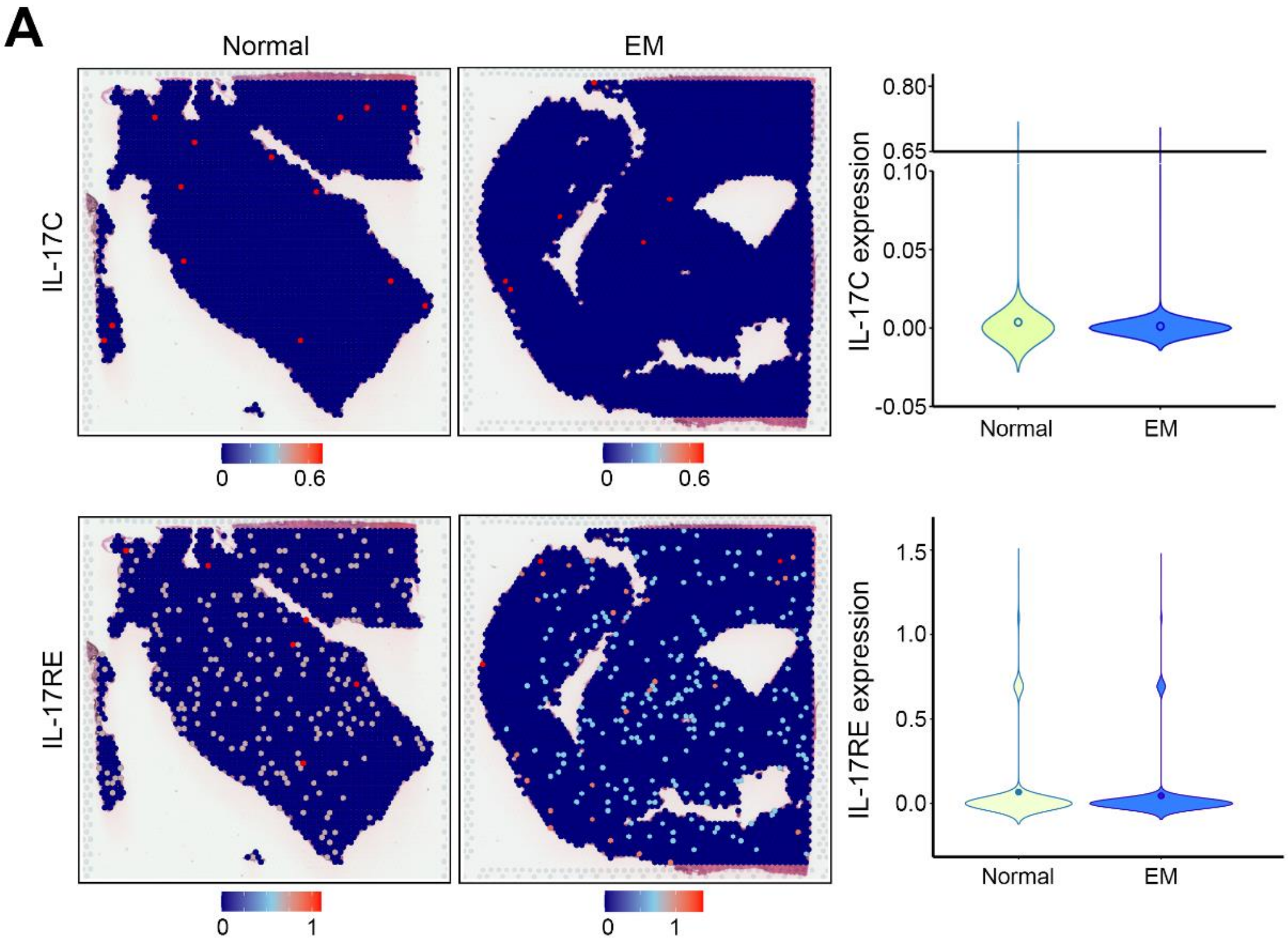

Supplementary Figure S8. Spatial distribution for the expression of IL-17C and IL-17RE.

A

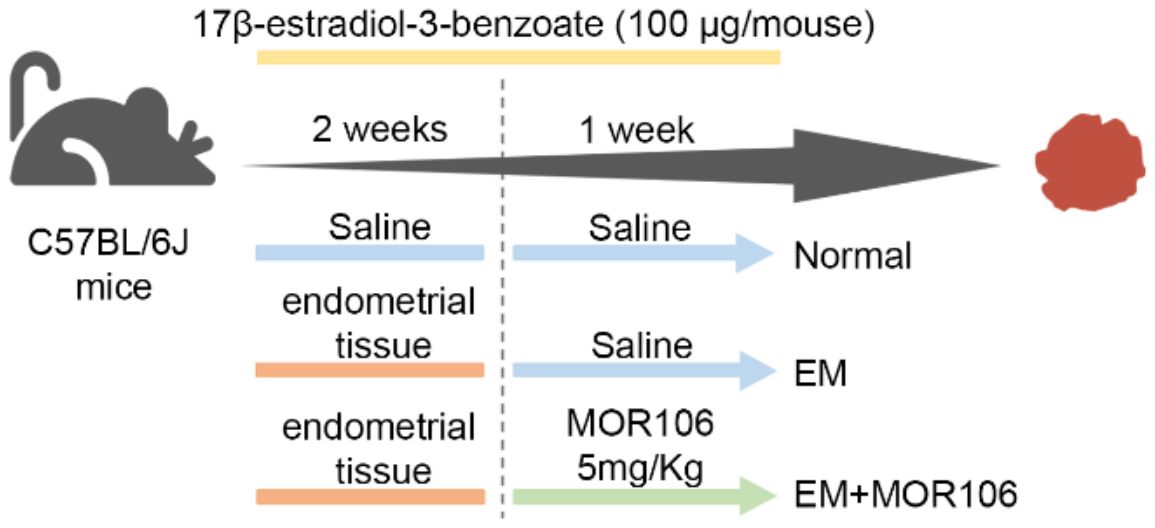

Supplementary Figure S9. Schematic diagram of a mouse model of endometriosis with or without MOR106 metformin treatment, showing the experimental groups and treatment timeline.

A

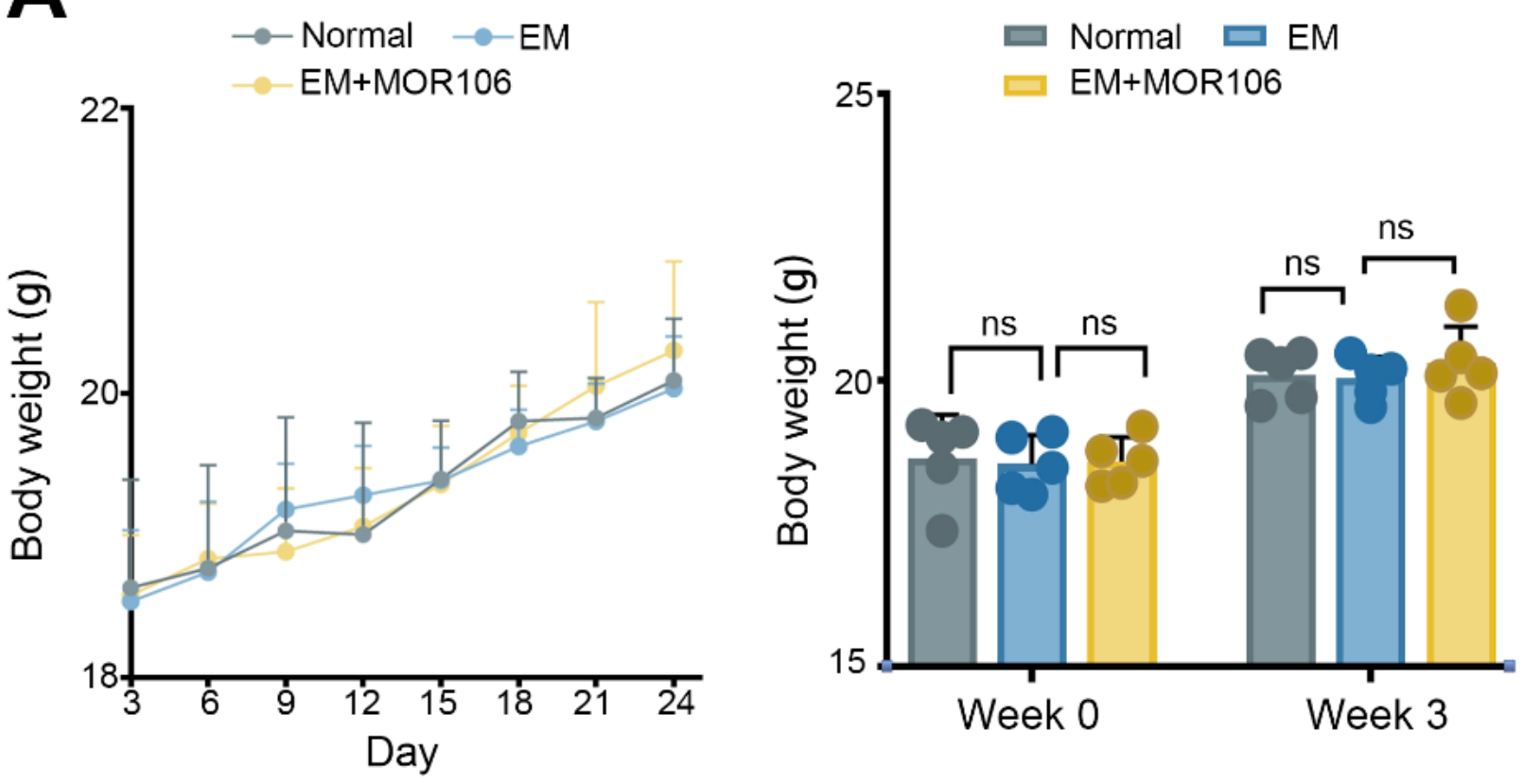

Supplementary Figure S10. Body weight of mice. n=5 animals per group. Each dot represents a mouse. Data represent the mean ± SD. 2-tailed Student's test. ns, not statistically significant.

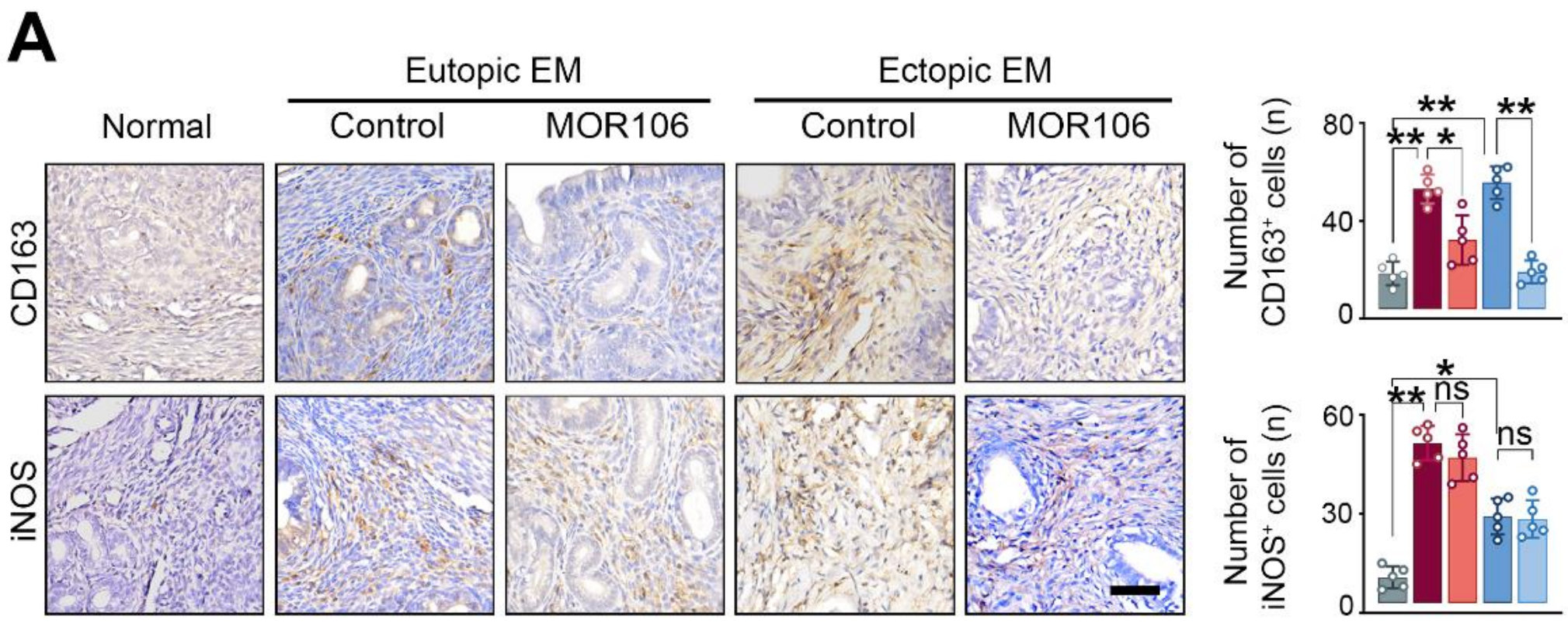

Supplementary Figure S11. The expression of CD163 and iNOS were detected by Immunostaining in EM mice. Scale bars: 50 µm. ns, not significant. *P<0.05 and **P<0.01 for the indicated comparisons.

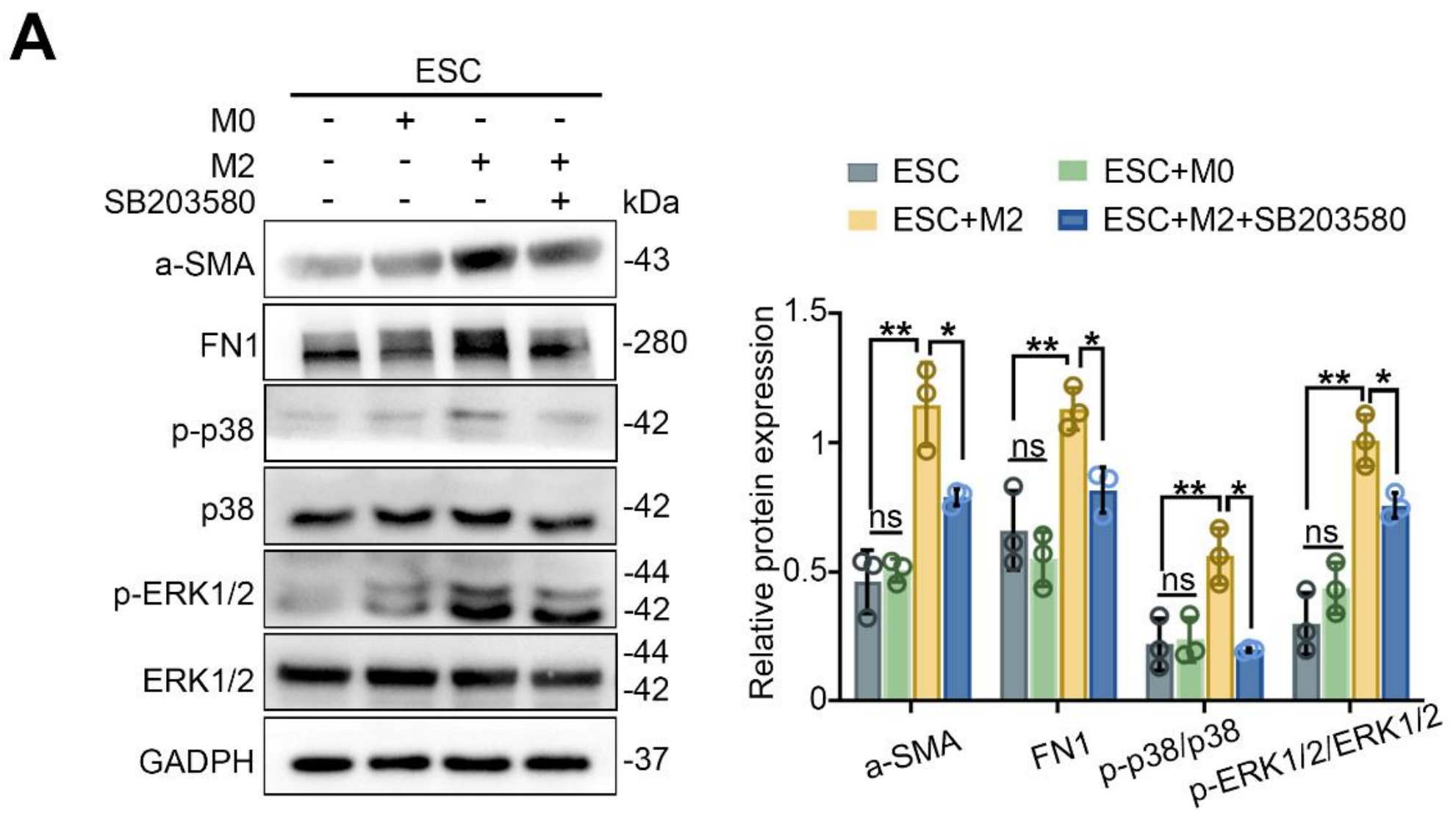

Supplementary Figure S12. Cells were treated with MAPK inhibitor under THP-1-ESC co-culture. (A) The expression of ECM-related and MAPK pathway key proteins was detected by western blot. ns, not significant. **P*<0.05 and ***P*<0.01 for the indicated comparisons.
